# Oral Astilbin Nanogels Modulate Senescence‐Associated CD4^+^ T Cells and Promote T_reg_ Induction in Aged Mice with Colitis

**DOI:** 10.1002/advs.78123

**Published:** 2026-10-18

**Authors:** Lei Zhao, Xinyun Zhang, Qian Ren, Xue Song, Meifang Zhao, Rongshen Wang, Haocheng Du, Yan Chen, Peigen Xu, Jiangyun Liu, Jian Zhang, Jingjing Ma

**Affiliations:** ^1^ Department of Pharmacy The Fourth Affiliated Hospital of Soochow University College of Pharmaceutical Sciences Medical Center of Soochow University Suzhou Jiangsu People's Republic of China; ^2^ Department of Pharmacy The Second Affiliated Hospital of Soochow University Suzhou Jiangsu People's Republic of China; ^3^ Laboratory Animal Center of Suzhou Medical College Soochow University Suzhou Jiangsu People's Republic of China; ^4^ Department of Hematology, The Affiliated Hospital of Qingdao University Qingdao University Qingdao Shandong People's Republic of China; ^5^ School of Pharmacy Shandong Second Medical University Weifang Shandong People's Republic of China; ^6^ School of Life Science and Technology Shandong Second Medical University Weifang Shandong People's Republic of China

**Keywords:** aged colitis, astilbin, CD4^+^ T cells, colon‐targeted delivery, oral nanogels, regulatory T cells

## Abstract

Inflammatory bowel disease (IBD) in older individuals is characterized by immune senescence and deteriorating intestinal homeostasis, both of which limit the efficacy of current therapies. Here, we develop fluorinated β‐glucan/pectin nanogels (F_2_‐Ast@NGs) for oral delivery of astilbin, a natural flavonoid with anti‐inflammatory and immunoregulatory activities. This multifunctional platform integrates gastrointestinal protection, colon‐targeted release, and local immune modulation. The β‐glucan shell preserves nanogel stability during upper gastrointestinal transit while enabling β‐glucanase‐responsive disassembly in the colon. Fluorocarbon segments enhance mucus penetration, and the pectin matrix prolongs colonic retention, promoting drug accumulation at inflamed sites. In aged mice with dextran sulfate sodium (DSS)‐induced colitis, F_2_‐Ast@NGs alleviate disease severity, attenuate senescence‐associated features of colonic CD4^+^ T cells, and enhance Foxp3^+^ regulatory T cell (T_reg_) responses. Concurrently, the β‐glucan/pectin nanogel matrix contributes to increased short‐chain fatty acid production and favorable alterations in gut microbial composition. Notably, the therapeutic efficacy of F_2_‐Ast@NGs is markedly attenuated in Foxp3^+^ T_reg_‐depleted mice, supporting an important role for T_reg_‐mediated immunoregulation. Collectively, F_2_‐Ast@NGs enable precise colonic delivery while coordinately remodeling the intestinal immune and microbial microenvironment, providing a promising oral nanotherapeutic strategy for colitis in aged hosts.

## Graphical abstract

Fluorinated β‐glucan/pectin nanogels enable colon‐targeted oral delivery of astilbin through gastrointestinal protection, β‐glucanase‐responsive release, mucus penetration, and prolonged retention. The released astilbin attenuates senescence‐associated features of CD4^+^ T cells and promotes T_reg_ responses, while the nanogel matrix modulates the gut microbiota and short‐chain fatty acids, collectively restoring intestinal immune homeostasis in aged mice with colitis.

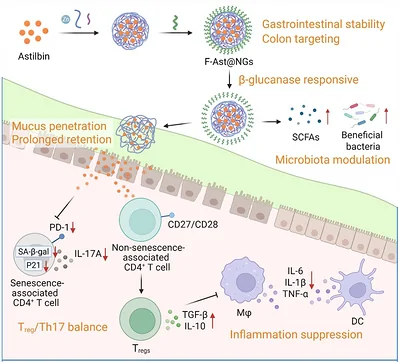

## 1 Introduction

Inflammatory bowel disease (IBD), encompassing ulcerative colitis (UC) and Crohn's disease (CD), is a chronic immune‐mediated disorder of the gastrointestinal (GI) tract characterized by recurrent intestinal inflammation and progressive impairment of quality of life [1, 2, 3]. As life expectancy continues to increase worldwide, the burden of IBD in older adults has expanded substantially. Elderly‐onset IBD, commonly defined as diagnosis at ≥ 60 years of age, has therefore emerged as an increasingly important clinical challenge [4, 5]. Epidemiological studies predict that more than one‐third of patients attending IBD clinics will be over 60 years old within the coming decade, reflecting the growing impact of population aging on IBD management [5, 6]. The aged intestinal microenvironment, characterized by impaired epithelial regeneration, microbial dysbiosis, and increased mucosal permeability, predisposes older individuals to aggravated intestinal injury [7, 8]. Consequently, compared with younger‐onset IBD, elderly patients exhibit more pronounced disruption of barrier integrity, heightened inflammation, and distinct dysbiosis in gut microbiota composition, all of which complicate disease management [9, 10, 11]. Moreover, immunosenescence and frailty further limit the use of immunosuppressive and biologic therapies owing to the increased risks of infection, malignancy, and poor drug tolerance [9, 12, 13]. Therefore, the management of colitis in aged individuals remains a major therapeutic challenge that requires attention and the development of more effective interventions.

Among the immune alterations driving IBD pathogenesis during aging, CD4^+^ T cells occupy a central position. Aging induces profound remodeling of the CD4^+^ T cell landscape, featuring thymic involution, contraction of the naïve T cell pool, and accumulation of senescent effector and effector memory subsets with hyperinflammatory phenotypes [14, 15]. Although aged CD4^+^ T cells exhibit impaired proliferative capacity and metabolic fitness, they secrete excessive proinflammatory cytokines, including IL‐17, thereby amplifying intestinal inflammation [14, 16, 17]. Recent murine studies have revealed that senescent CD4^+^ T cells represent the predominant immune population with elevated senescence‐associated β‐galactosidase (SA‐β‐gal) activity in aged colonic tissues and contribute substantially to IL‐17 production during dextran sulfate sodium (DSS)‐induced colitis [16]. Furthermore, dysfunctional CD4^+^ T cells disrupt the homeostatic balance between effector and regulatory T cells (T_regs_), impair mucosal healing, and contribute to loss of gut barrier integrity and microbiota dysbiosis [15, 18, 19]. Importantly, senescent CD4^+^ T cells commonly display elevated expression of programmed death‐1 (PD‐1), a co‐inhibitory receptor whose sustained activation suppresses T cell differentiation potential and contributes to functional exhaustion [20, 21]. Overall, these findings identify senescent CD4^+^ T cells as pivotal mediators linking immunosenescence to intestinal inflammation, highlighting them as promising targets for T cell‐directed therapies in aged colitis.

Astilbin (Ast), a flavonoid glycoside extracted from *Smilax glabra Roxb*., has attracted considerable interest for its anti‐inflammatory activities. Pharmacological studies have demonstrated that Ast can effectively alleviate a variety of inflammatory disorders such as rheumatoid arthritis, systemic lupus erythematosus, liver injury, IBD, and metabolic disorders including diabetes, primarily by suppressing the NF‐κB and PI3K/AKT signaling pathways and regulating the activation of multiple immune cell subsets [22, 23, 24, 25, 26]. Beyond its anti‐inflammatory effects, recent research suggests that Ast also possesses anti‐senescence properties, partly through the reduction of oxidative stress and restoration of mitochondrial homeostasis, thereby mitigating aging‐related degeneration [27]. Moreover, Ast has been shown to rebalance immune homeostasis by increasing T_regs_ and reducing proinflammatory Th17 cells, effectively controlling chronic inflammation [28, 29, 30]. Despite these promising therapeutic profiles, the clinical application of Ast remains hampered by its extremely low oral bioavailability (< 1%), even at high doses of 40–80 mg kg^−1^, as corroborated by our earlier findings [22, 23, 24, 31]. This limitation arises from multiple physicochemical and pharmacokinetic barriers: (i) poor aqueous solubility, (ii) chemical instability within the GI tract, and (iii) low intestinal absorption efficiency [24, 25]. Therefore, improving the GI stability and intestinal absorption of Ast is imperative to fully harness its therapeutic potential.

Given the prominent roles of immune senescence and CD4^+^ T cell imbalance in aged colitis, together with the limited gastrointestinal stability and bioavailability of Ast, there is a need to develop a delivery strategy that enhances its therapeutic potential. However, whether Ast can modulate aging‐associated CD4^+^ T cell dysfunction, particularly senescence‐associated phenotypes, in aged colitis remains unclear. In this work, we engineered fluorinated β‐glucan/pectin‐based nanogels (F‐Ast@NGs) that efficiently encapsulate Ast and preserve its stability during gastrointestinal transit for aging‐related colitis treatment (Scheme 1). The β‐glucan shell improves the stability of the NGs under the acidic and bile salt conditions of the upper gastrointestinal tract [32], while enabling microbiota‐responsive disassembly in the colon [33, 34], where microbial β‐glucanases trigger site‐specific Ast release. The fluorocarbon modification altered the structure of the NGs and markedly enhanced their mucus penetration capability. In addition, pectin incorporation conferred excellent colonic mucoadhesive properties through hydrogen bonding and ionic interactions with mucin components, enabling prolonged colonic retention [35, 36]. Mechanistically, in aged DSS‐induced colitis mice, the released Ast reshaped colonic CD4^+^ T cell profiles by reducing senescence‐associated CD4^+^ T cells and increasing CD25^+^ Foxp3^+^ T_regs_. Further analysis showed that Ast preferentially suppressed the metabolic activity and pro‐inflammatory phenotype of senescence‐associated CD4^+^ T cells while promoting T_reg_ differentiation from non‐senescence‐associated CD4^+^ T cells. This shift toward a more regulatory immune environment was accompanied by increased TGF‐β and IL‐10 levels and reduced production of pro‐inflammatory cytokines, including IL‐17A, TNF‐α, IL‐6, and IL‐1β. Meanwhile, the NGs system elevated the levels of short‐chain fatty acids (SCFAs) in the GI tract and reshaped gut microbiota composition by enriching beneficial genera, including *Lactobacillus*, *Lachnospiraceae_NK4A136_group*, and *Odoribacter*. Altogether, this study establishes a colonic targeted nanotherapeutic system that attenuates senescence‐associated features of CD4^+^ T cells, rebalances T_reg_/Th17 homeostasis, and restores intestinal immune and microbial equilibrium, offering a promising therapeutic strategy for colitis in aged hosts.

**SCHEME 1:**
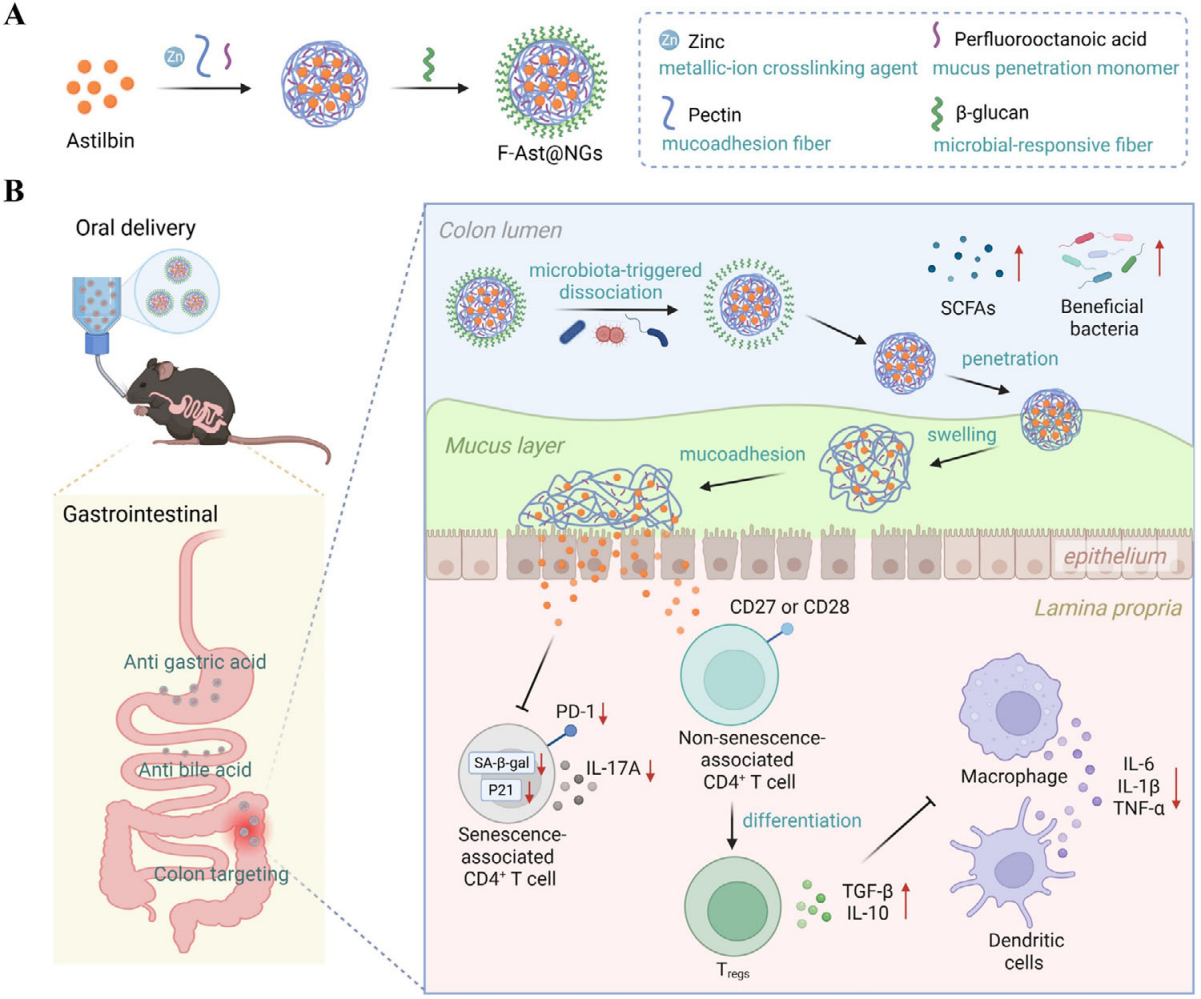
Schematic illustration of the fabrication and therapeutic mechanism of F‐Ast@NGs for IBD treatment. (A) Preparation process of F‐Ast@NGs. (B) After oral administration, F‐Ast@NGs alleviate colitis by inhibiting senescence‐associated CD4^+^ T cells and restoring intestinal immune and microbial homeostasis. Created with BioRender.

## 2 Results and Discussion

### 2.1 Aging Exacerbates Colitis Through Senescence‐Associated Dysfunction of CD4^+^ T Cells

PD‐1, an immune checkpoint receptor involved in T cell exhaustion and chronic immune regulation, has been reported to be elevated in dysfunctional and senescence‐associated T cell populations. Although PD‐1 is not a specific marker of cellular senescence, its expression may reflect a senescence‐associated functional state of CD4^+^ T cells [37, 38]. We first evaluated the relationship between *PDCD1* expression, which encodes PD‐1, and age in patients with colitis using publicly available transcriptomic data from the Gene Expression Omnibus (GEO) database. *PDCD1* expression exhibited a positive correlation with age, suggesting an age‐associated increase in *PDCD1* expression (Figure 1A). Furthermore, patients were stratified into young (< 60 years) and aged (≥ 60 years) groups based on the clinical definition of elderly‐onset IBD [5]. The aged cohort displayed significantly higher *PDCD1* expression levels than the young cohort (Figure 1B).

**FIGURE 1:**
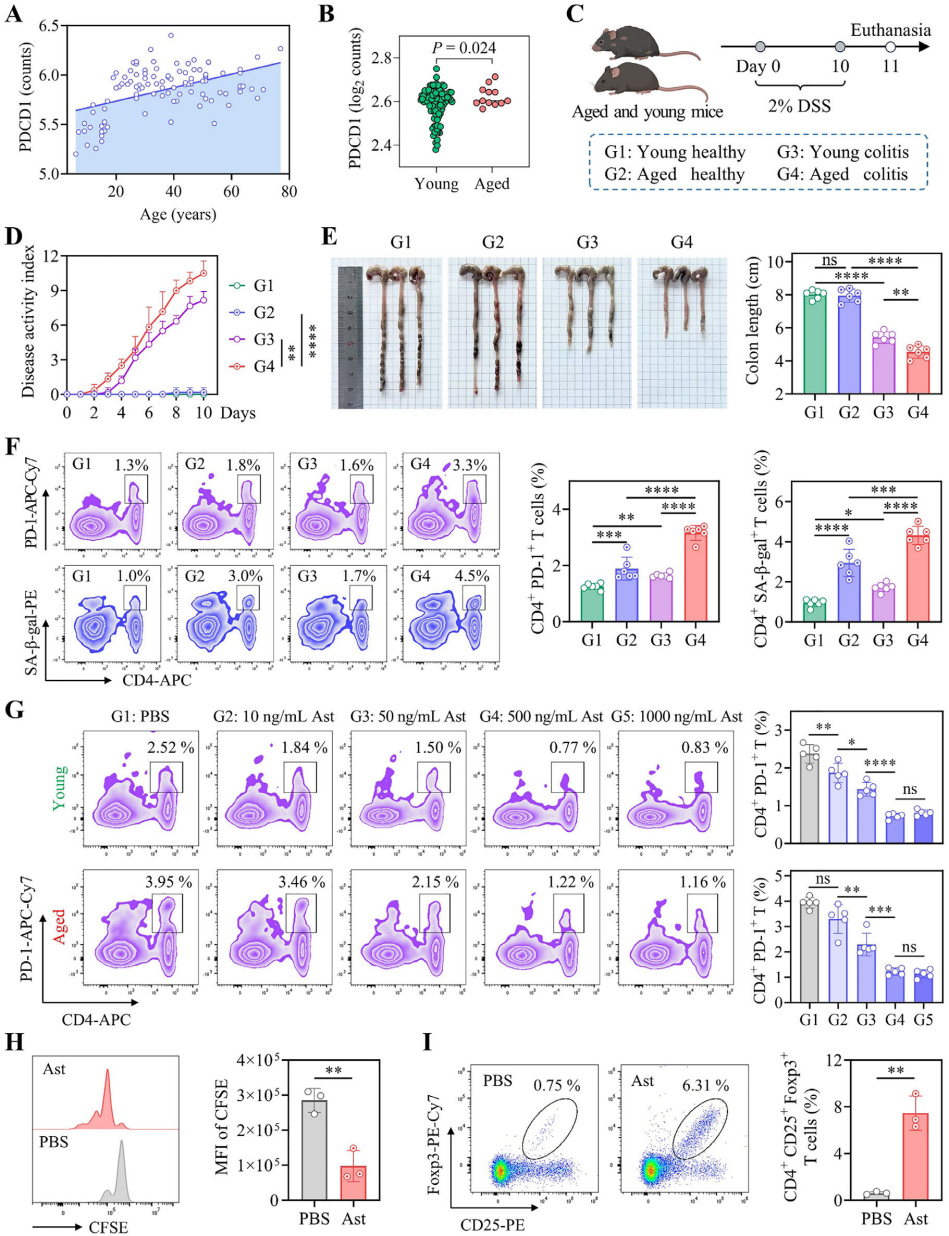
Ast attenuates senescence‐associated features and restores the immunoregulatory function of CD4^+^ T cells. (A) Correlation between *PDCD1* expression, which encodes PD‐1, and the age at diagnosis in patients with colitis (*n* = 106). (B) Comparison of *PDCD1* expression levels between young (< 60 years old) and aged (≥ 60 years old) colitis patients. (C) Experimental flowchart for DSS‐induced colitis in young and aged mice. (D) Curve chart analysis of DAI (*n* = 6). (E) Representative images of colons and corresponding quantification of colon length (*n* = 6). (F) Quantitative analysis of CD4^+^ PD‐1^+^ T cells and CD4^+^ SA‐β‐gal^+^ T cells in colonic lamina propria cells and representative FC plots (*n* = 6). (G) In vitro effects of different concentrations of Ast on PD‐1 expression in colonic CD4^+^ T cells isolated from young and aged mice (*n* = 5). (H) In vitro proliferation of CD4^+^ T cells isolated from aged mice following Ast treatment (*n* = 3). (I) In vitro differentiation of CD4^+^ T cells isolated from aged mice into T_regs_ following Ast treatment (*n* = 3). Data are presented as mean ± SD and analyzed by two‐tailed unpaired Student's t test with Welch's correction (B), two‐tailed unpaired Student's t test (H, I), Two‐way ANOVA with Tukey's post‐test (D), or One‐way ANOVA with Tukey's post‐test (E–G). ns represents not significant, ^*^ *P* < 0.05, ^**^ *P* < 0.01, ^***^ *P* < 0.001, and ^****^ *P* < 0.0001.

To further explore the impact of aging on colitis progression, we compared disease severity between young and aged colitis mice (Figure 1C). On day 11, both young and aged colitis mice exhibited severe hematochezia (Figure S1A), implying substantial intestinal injury. Both colitis groups showed a marked reduction in body weight, while aged colitis mice displayed a greater body weight loss than young colitis mice (Figure S1B). Consistent with these findings, aged colitis mice exhibited significantly elevated disease activity index (DAI) compared with younger mice, as evaluated by changes in body weight, stool characteristics, and rectal bleeding (Figure 1D). Although the colon length of healthy aged mice was comparable to that of young healthy mice, the colon length of aged colitis mice (4.5 cm) was markedly shorter than that of young colitis mice (5.4 cm) (Figure 1E), indicating that aged mice are more susceptible to colitis progression. Furthermore, the colonic IL‐17A concentration was significantly higher in aged colitis mice (Figure S1C). Histopathological analysis by hematoxylin and eosin (H&E) staining revealed extensive epithelial damage and massive infiltration of inflammatory cells in both colitis groups, with aged colitis mice exhibiting the highest histological score among all groups (Figure S1D). To determine whether colitis affects PD‐1 expression in mice, colonic lamina propria cells were isolated for flow cytometry (FC) analysis. As expected, PD‐1 expression on colonic CD4^+^ T cells was markedly higher in aged mice than in young mice, and colitis further substantially enhanced PD‐1 expression, especially in aged mice (Figure 1F). Moreover, the activity of SA‐β‐gal and the expression of P21, two widely used markers of cellular senescence [39], were significantly upregulated in colonic CD4^+^ T cells from aged mice compared with those from young mice, and were further increased after colitis induction (Figure 1F and Figure S1E), suggesting that aging and colitis synergistically promote senescence‐associated changes in CD4^+^ T cells. Following DSS induction, the proportion of T_regs_ in the colon increased 3.4 fold in young mice, likely to counteract inflammation, whereas the increase was only 1.7 fold in aged mice (Figure S1F), suggesting that aging may compromise the compensatory T_reg_ response during colonic inflammation.

To assess the functional contribution of colonic CD4^+^ T cells to colitis progression, CD4^+^ T cells isolated from young and aged mice, either healthy or with colitis, were adoptively transferred into colitis recipient mice on day 2 (Figure S2A). All recipient mice developed serious hematochezia after DSS induction (Figure S2B). Notably, colitis mice receiving CD4^+^ T cells derived from aged colitis donors displayed the most severe disease phenotype, characterized by the lowest body weight, highest DAI, shortest colon length, and highest colonic IL‐17A levels among all groups (Figure S2C–F).

Taken together, these findings indicate that colonic CD4^+^ T cells play an important role in colitis pathogenesis, and that aging and colitis are associated with enhanced senescence‐associated features and PD‐1 upregulation in CD4^+^ T cells.

### 2.2 Ast Attenuates Senescence‐Associated Features in CD4^+^ T Cells and Promotes T_reg_ Induction

To evaluate whether Ast could attenuate aging and DSS‐induced PD‐1 upregulation in CD4^+^ T cells, colonic cells isolated from aged and young mice (healthy or colitis) were treated with Ast. The results showed that Ast significantly reduced PD‐1 expression on CD4^+^ T cells from both aged and young colitis mice (Figure S3). Furthermore, concentration response analysis showed that 500 ng mL^−1^ of Ast produced maximal suppression of PD‐1, with no further inhibition at higher concentrations (Figure 1G and Figure S4). To further assess the functional status of Ast‐treated senescence‐associated CD4^+^ T cells, cells were incubated with Ast and analyzed for senescence‐ and function‐related readouts. Ast treatment significantly decreased P21 expression in CD4^+^ T cells (Figure S5). In parallel, Ast promoted CD4^+^ T cell proliferation (Figure 1H) and enhanced Foxp3^+^ T_reg_ induction (Figure 1I). Collectively, these results indicate that Ast reduces senescence‐associated CD4^+^ T cell features and favors a T_reg_ response.

The therapeutic efficacy of free Ast was next examined in aged and young colitis mice (Figure 2A). Following oral administration, only high‐dose Ast (100 mg kg^−1^) exhibited significant therapeutic benefits, as evidenced by alleviated hematochezia, improved body weight maintenance, decreased DAI, and restoration of colon length compared with the low‐dose group (20 mg kg^−1^) (Figure 2B–D and Figure S6). In aged colitis mice, the high‐dose Ast achieved moderate efficacy, whereas the low‐dose was largely ineffective. FC analysis revealed that high‐dose Ast markedly decreased the proportion of colonic CD4^+^ PD‐1^+^ T cells (Figure 2E), accompanied by a decrease in CD4^+^ SA‐β‐gal^+^ and CD4^+^ P21^+^ T cells and an increase in the frequency of T_regs_ (Figure 2F, G and Figure S7).

**FIGURE 2:**
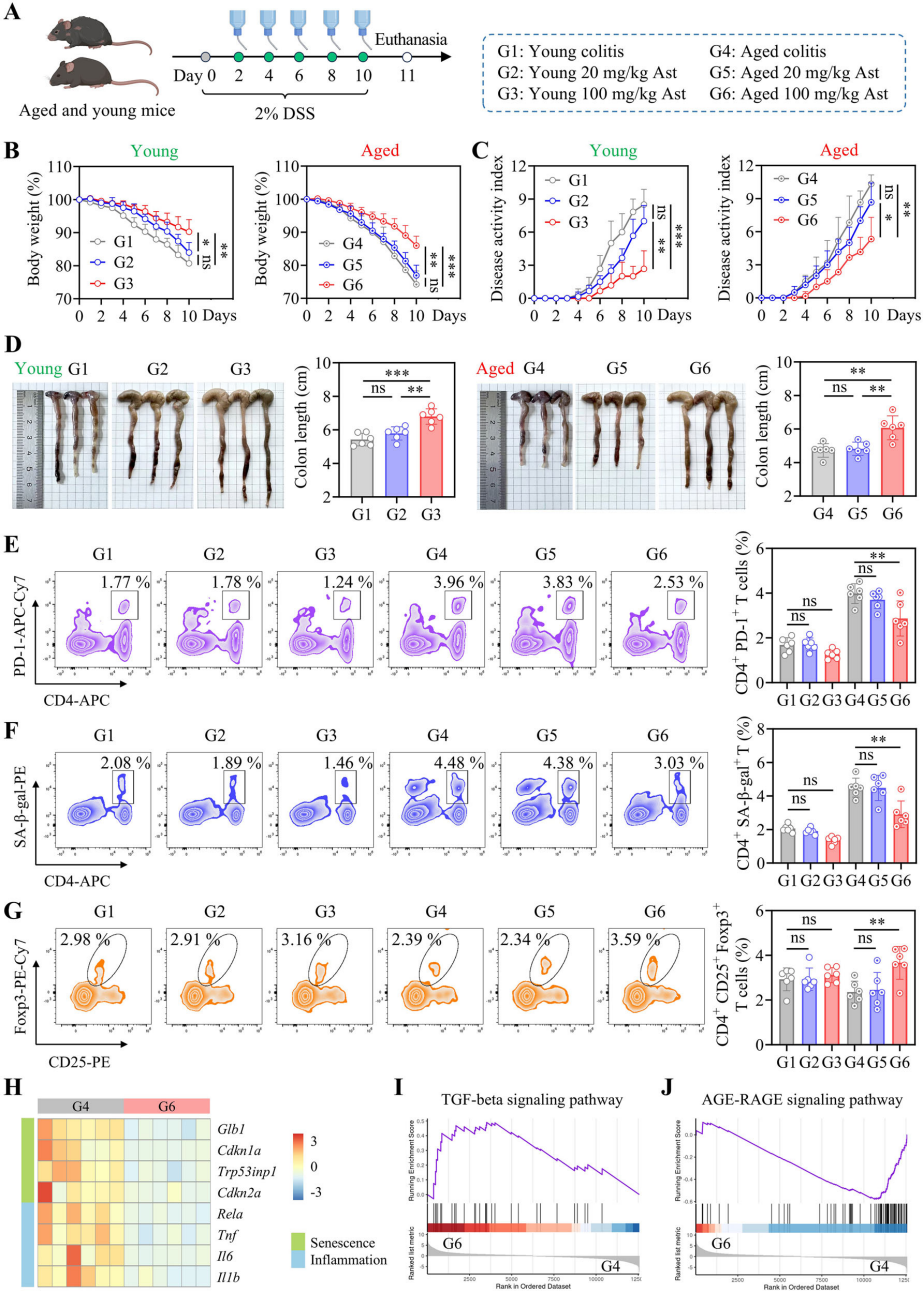
High‐dose free Ast alleviates colitis in young and aged mice by attenuating senescence‐associated immune dysfunction. (A) Experimental flowchart of young and aged colitis mice treated with free Ast. (B) Curve chart variations of murine body weight across time, normalized to the day 0 body weight percentage (*n* = 6). (C) Curve chart analysis of DAI (*n* = 6). (D) Representative images of colons and corresponding quantification of colon length in young and aged mice (*n* = 6). Representative FC plots and quantitative analysis of (E) CD4^+^ PD‐1^+^ T cells, (F) CD4^+^ SA‐β‐gal^+^ T cells, and (G) CD25^+^ Foxp3^+^ CD4 T cells in colonic lamina propria cells (*n* = 6). (H) Heatmap of differentially expressed genes in colon samples (*n* = 6). GSEA of the (I) TGF‐beta signaling pathway and (J) AGE‐RAGE signaling pathway. Data are presented as mean ± SD and analyzed by One‐way ANOVA with Tukey's post‐test (D‐G) or Two‐way ANOVA with Tukey's post‐test (B, C). ns represents not significant, ^*^ *P* < 0.05, ^**^ *P* < 0.01, and ^***^ *P* < 0.001.

To investigate the molecular mechanisms of Ast‐mediated therapeutic effects, high‐throughput RNA sequencing was performed on colon tissues from aged colitis mice. Principal component analysis (PCA) showed that gene expression profiles of the high‐dose Ast group closely resembled those of healthy controls, whereas untreated colitis mice exhibited distinct transcriptomic signatures (Figure S8A). Key senescence‐associated genes, including *Glb1*, *Cdkn1a*, *Trp53inp1* and *Cdkn2a*, which regulate lysosomal activity, cell cycle arrest and p53‐mediated stress responses, were markedly downregulated following Ast treatment (Figure 2H). Similarly, pro‐inflammation‐related genes such as *Rela*, *Tnf*, *Il6* and *Il1b* were reduced (Figure 2H), whereas anti‐inflammation‐related genes including *Tgfb1*, *Il10* and *Foxp3* were notably elevated after Ast treatment (Figure S8B). Gene Ontology (GO) enrichment analysis indicated that Ast treatment modulated pathways related to inflammation and oxidative stress, including “hydrogen peroxide catabolic process” and “reactive oxygen species metabolic process” (Figure S8C), suggesting enhanced antioxidant capacity. Enrichment of “erythrocyte development” and “heme binding” further implies improved redox balance, while “fucosylation” and related biosynthetic processes indicate modulation of inflammation‐associated senescence, collectively contributing to mucosal recovery in colitis. Furthermore, KEGG‐based gene set enrichment analysis (GSEA) identified the “TGF‐beta” signaling pathway as one of the significantly enriched pathways in the Ast group (Figure 2I), indicating suppression of excessive inflammatory activation. Conversely, the “AGE‐RAGE” signaling pathway, closely associated with oxidative stress and aging, was markedly enriched in the DSS‐treated group (Figure 2J), further underscoring the anti‐inflammatory effects of Ast and its modulation of aging‐associated processes.

These results indicate that Ast attenuates senescence‐associated features of CD4^+^ T cells, reduces the proportion of CD4^+^ PD‐1^+^ T cells and increases T_reg_ abundance, thereby contributing to improved immune homeostasis in colitis, particularly in aged mice.

### 2.3 Characterization and β‐Glucanase‐Responsive Release of F‐Ast@NGs

In our previous study, free Ast exhibited poor stability in the GI tract [24], requiring a high oral dose to achieve a therapeutic effect. To overcome this limitation, we developed an oral NG formulation to enhance its GI stability. The NG core was fabricated through electrostatic interaction, hydrophobic interaction, and coordination interaction among Ast, zinc ions, pectin, and perfluorooctanoic acid (PFOA), followed by β‐glucan coating via hydrogen bonding and van der Waals interactions to yield F_x_‐Ast@NGs (Scheme 1A). The numerical designation x (1–4) denotes PFOA monomer contents of 1, 2, 3, and 4 wt.%, respectively (Table S1). Non‐fluorinated Ast@NGs were generated using an identical protocol, except that PFOA was omitted during preparation. The hydrodynamic diameter of Ast@NGs was 579 nm, while that of F‐Ast@NGs gradually increased from 628 to 871 nm with increasing PFOA content (Figure 3A), attributed to the reduced compactness of the NG network introduced by hydrophobic fluorocarbon chains. Transmission electron microscopy (TEM) characterization revealed that all NGs exhibited a spherical morphology with an integrated structure, and their diameters ranged from 400 to 600 nm (Figure 3B). Zeta potential measurements revealed that increasing PFOA content decreased the surface potential of F‐Ast@NGs (Figure 3C), likely due to the higher proportion of carboxyl groups in PFOA. Ast was efficiently encapsulated inside the NGs, achieving over 80% loading efficiency (Figure S9A). The PDI values of Ast@NGs and F‐Ast@NGs were within 0.3 (Figure S9B), reflecting high homogeneity and good colloidal stability.

**FIGURE 3:**
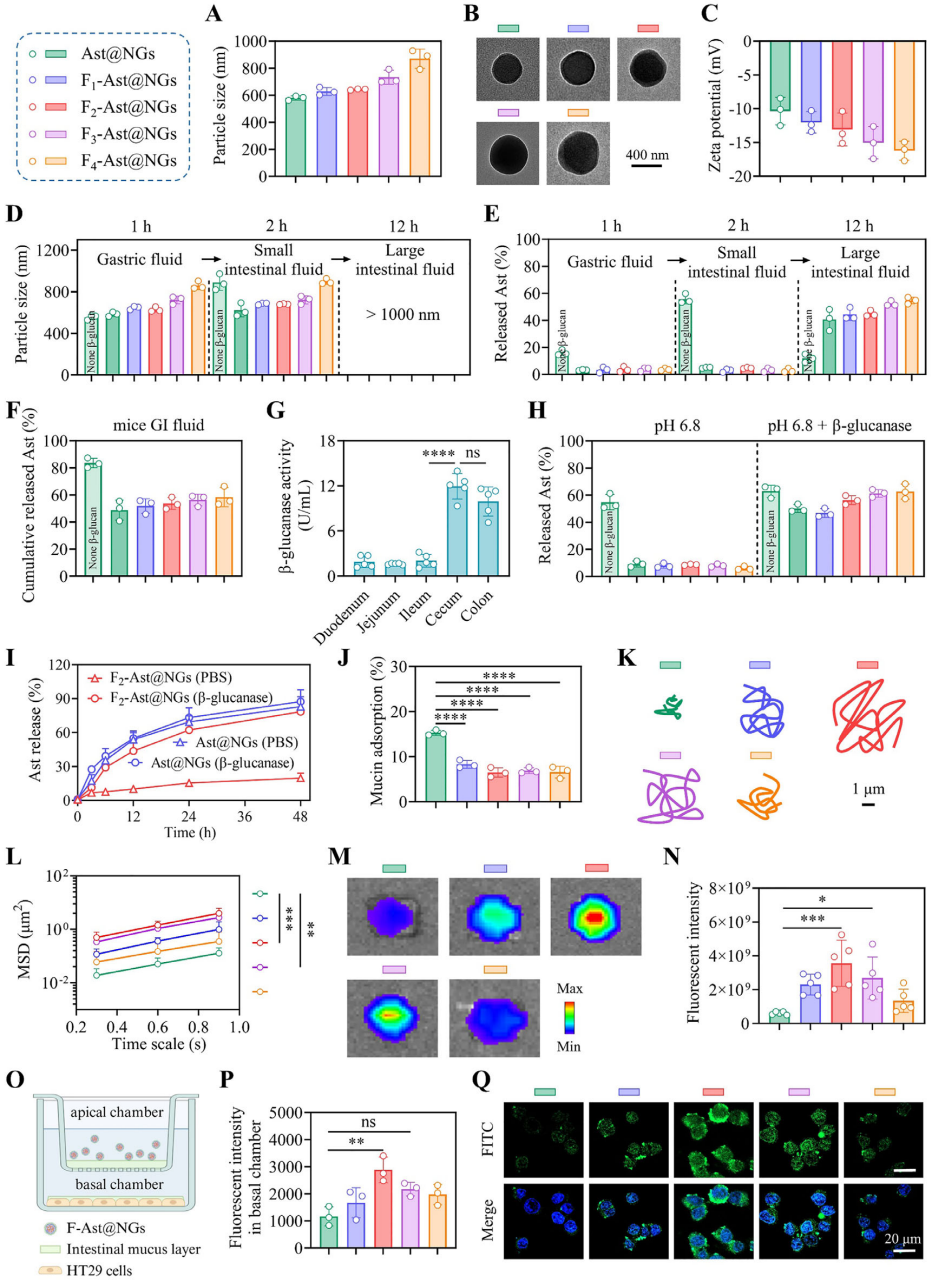
β‐glucanase‐responsive and mucus penetration of F‐Ast@NGs. (A) Particle size of NGs (*n* = 3). (B) Representative TEM images (Scale bar = 400 nm). (C) Zeta potential of NGs (*n* = 3). (D) Changes in particle size of NGs after incubation with mice GI fluid (*n* = 3). (E) Released content of Ast from NGs in mice GI fluid (*n* = 3). (F) Cumulative Ast release in mouse GI fluid (*n* = 3). (G) β‑glucanase activity in mouse duodenum, jejunum, ileum, cecum, and colon (*n* = 5). (H) Released content of Ast from NGs in the presence of β‑glucanase (*n* = 3). (I) Release profile of β‐glucan‐uncoated Ast@NGs and F_2_‐Ast@NGs in PBS or β‐glucanase solution (*n* = 3). (J) The proportion of NGs that formed aggregates with mucin (*n* = 3). (K) Representative trajectories of NGs in the mucus during the 10 s movies. (L) Ensemble‐averaged MSD of NGs as a function of the time scale (τ) (*n* = 3). (M) Representative IVIS images of colon segments after NGs penetration. (N) FI of colon pieces (*n* = 5). (O) Schematic illustration of mucus penetration setup. Created with BioRender. (P) FI of the medium in the basal chamber (*n* = 3). (Q) CLSM images of HT29 cells after 6 h of incubation with various NGs (Scale bar = 20 µm). Data are presented as mean ± SD and analyzed by One‐way ANOVA with Tukey's post‐test (G, J, N, P) or Two‐way ANOVA with Tukey's post‐test (L). ns represents not significant, ^*^ *P* < 0.05, ^**^ *P* < 0.01, ^***^ *P* < 0.001, and ^****^ *P* < 0.0001.

Because orally administered nanocarriers are exposed to pronounced pH changes along the GI tract, the stability of the NGs was evaluated under sequential pH conditions. The suspension was first adjusted from pH 7.4 to 1.2 to mimic the gastric environment and was subsequently returned to pH 6.8 to represent intestinal conditions. β‐glucan‐coated NGs showed negligible changes in particle size throughout this process, while Ast release remained below 15% (Figure S10). In contrast, uncoated Ast@NGs exhibited pronounced swelling to ∼925 nm at pH 6.8 and experienced extensive Ast leakage (∼50.6%). These findings suggested that the β‐glucan shell effectively maintained NG stability during pH fluctuations. Ulteriorly, we examined the stability in bile salts. β‐glucan‐coated NGs exhibited goodtolerance to 4% bile salts, maintaining a relatively stable particle size and effectively retaining the encapsulated Ast compared with uncoated Ast@NGs (Figure S11). The favorable protective effect was expected, given the presence of the β‐glucan shell, which hindered the penetration of bile salts into the NGs. To better assess GI stability, NGs were incubated with mouse‐derived GI fluids. The diameter of β‐glucan‐coated NGs was stable in gastric fluid and small intestinal fluid, whereas uncoated Ast@NGs significantly expanded in the small intestine (Figure 3D). Interestingly, all NGs exhibited diameters over 1000 nm in large intestinal fluid. Correspondingly, uncoated Ast@NGs displayed continuous Ast release at all stages, particularly in small intestinal fluid (> 55%), whereas β‐glucan‐coated NGs released Ast primarily in large intestinal fluid (Figure 3E). The cumulative Ast release exceeded 80% for uncoated Ast@NGs compared with approximately 50% for β‐glucan‐coated formulation (Figure 3F).

To elucidate the release mechanism, β‐glucanase activity was examined across different intestinal regions. Consistent with the above observations, β‑glucanase exhibited remarkable activity in the cecum and colon, which were the principal sections of the large intestine (Figure 3G). Therefore, NGs were kept in a pH 6.8 solution with or without β‑glucanase. As expected, β‐glucan‐coated NGs showed considerable release of Ast under a β‑glucanase environment, which was similar to that of uncoated Ast@NGs (Figure 3H), implying enzyme‐mediated degradation of the β‐glucan shell. The β‐glucanase‐responsive release behavior was further evaluated using F_2_‐Ast@NGs, which released < 20% Ast within 48 h in PBS but ≈ 78% in the presence of β‐glucanase (Figure 3I). In contrast, uncoated Ast@NGs exhibited sustained, non‐responsive release irrespective of enzyme presence.

Together, these findings indicate that the β‐glucan shell effectively retains Ast within the nanogel matrix during transit through the upper GI tract, thereby minimizing premature drug release and allowing β‐glucanase‐responsive release in the colon.

### 2.4 Mucus Penetration and Mucoadhesion of F‐Ast@NGs

The dense mucus layer covering the intestinal epithelium acts as a physical barrier that hinders drug diffusion and nanoparticle penetration, ultimately reducing local drug accumulation and therapeutic efficacy. To probe the mucus penetration capability of F‐Ast@NGs, their interactions with mucin were first assessed using a mucin aggregation assay. All F‐Ast@NGs illustrated significantly reduced mucin adsorption compared with Ast@NGs (Figure 3J). To evaluate mucus transport, the movement of NGs in freshly isolated mouse intestinal mucus was analyzed via multiple particle tracking. Compared with Ast@NGs, all F‐Ast@NGs showed enhanced diffusion, and F_2_‐Ast@NGs and F_3_‐Ast@NGs displayed comparable and the largest diffusion areas (Figure 3K). Analysis of the ensemble‐averaged mean squared displacement (MSD) further supported the superior mucus mobility of F‐Ast@NGs, as their MSD values consistently exceeded those of Ast@NGs throughout the entire observation period (Figure 3L). Among all formulations, F_2_‐Ast@NGs exhibited the highest MSD values, indicating the strongest mucus mobility and penetration capability. Ex vivo colon tissue experiments further validated these results. Different FITC‐labeled NGs were incubated with colon segments, followed by PBS washing to remove unbound particles. The fluorescence intensity (FI) of all F‐Ast@NGs groups was higher than that of Ast@NGs (Figure 3M), indicating enhanced mucus retention. Quantitatively, FI of F_1_‐, F_2_‐, F_3_‐, and F_4_‐Ast@NGs was 3.8, 5.5, 4.4, and 2.2 times higher than that of Ast@NGs (Figure 3N). Based on this, the penetration ability of F‐Ast@NGs was investigated in a mucus‐producing intestinal barrier (Figure 3O). After 6 h of incubation, FI of the basal chamber medium in the F_2_‐Ast@NGs group was the highest among all groups (Figure 3P), confirming their superior mucus penetration ability. As a soluble dietary fiber, pectin possesses excellent colonic mucoadhesive properties through the formation of hydrogen bonds and ionic bridges with mucin components [35, 36]. F_2_‐Ast@NGs exhibited the highest FI in HT29 cells (Figure 3Q), indicating its effective colonic mucoadhesion mediated by the pectin.

Taken together, these results suggest that the incorporation of fluorocarbon moieties substantially enhances the mucus‐penetrating capability of NGs, which is likely attributable to the hydrophobic and lipophobic characteristics of fluorocarbons that minimize mucin glycoprotein adsorption on the particle surface. Among all formulations, F_2_‐Ast@NGs exhibited the highest MSD values and superior performance in *ex vivo* mucus retention, mucus penetration, and mucoadhesion assays. Therefore, considering the overall properties of mucus interaction, F_2_‐Ast@NGs were selected as the optimized formulation for subsequent in vivo evaluations.

### 2.5 Enhanced Retention of F_2_‐Ast@NGs in Inflamed Colon

Cy5‐labeled F_2_‐Ast@NGs or Ast@NGs were orally administered to colitis mice, and the entire intestinal tract was collected at different time points for IVIS imaging. Free Cy5 illustrated rapid gastrointestinal elimination following oral administration, with obvious FI in the proximal and distal intestinal regions, including the duodenum, jejunum, ileum, and cecum at 3 h, followed by only transient retention in the colon at 6 h (Figure 4A). In contrast, extended GI retention was observed in both NGs, particularly in F_2_‐Ast@NGs with effective colon retention for up to 48 h. Quantitative analyses across intestinal regions revealed that F_2_‐Ast@NGs were prominently distributed in the large intestine (cecum and colon), where their levels were substantially higher than those detected in the duodenum, jejunum, and ileum (Figure 4B and Figure S12). F_2_‐Ast@NGs exhibited markedly enhanced colon targeting compared with Ast@NGs, with FI levels 1.2, 2.0, and 2.3 fold higher at 12, 24, and 48 h, respectively (Figure 4B). Moreover, the area under the curve (AUC) of F_2_‐Ast@NGs in the colon was 1.8 fold higher than that of Ast@NGs (Figure S13), indicating promising accumulation in colon tissue. To directly visualize the penetration of NGs into the colonic mucus layer, frozen colon sections were prepared and stained with DAPI. After 6 h of administration, free Cy5 and Cy5‐labeled Ast@NGs were distributed only in the lumen of the colon (Figure 4C). Consistent results were also noted from colon sections at 48 h, where Ast@NGs showed limited mucosal penetration, whereas F_2_‐Ast@NGs displayed substantial distribution throughout the colonic mucosa (Figure 4D). The colon tissues were further subjected to 3D CLSM scanning. F_2_‐Ast@NGs penetrated deeply through the mucus layer, whereas such penetration was not observed with Ast@NGs (Figure 4E). Together, the findings indicate that fluorinated NGs achieved sustained colonic retention while readily traversing the mucus barrier, resulting in enhanced delivery and tissue accumulation in the colon.

**FIGURE 4:**
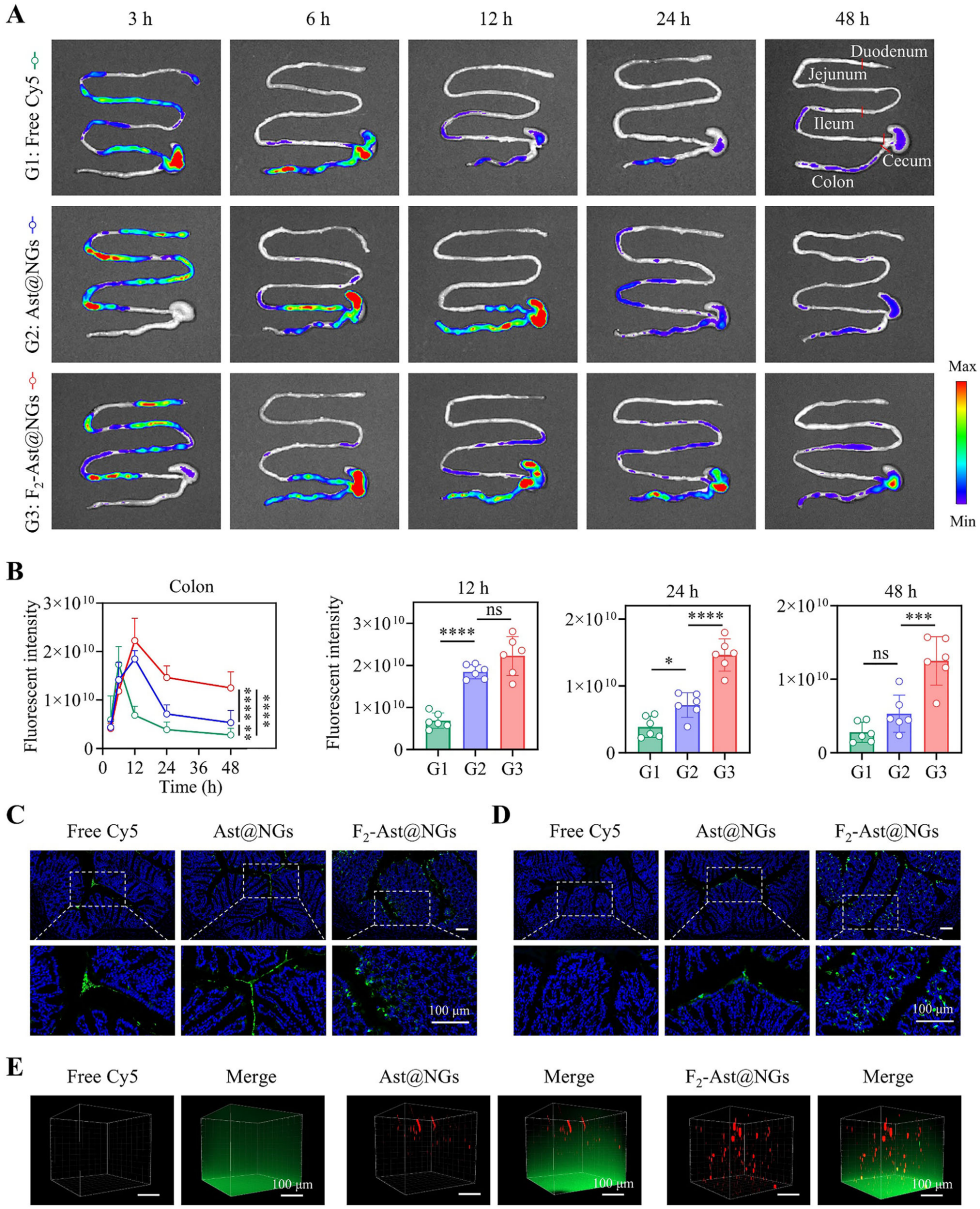
Enhanced colon retention and intestinal barrier penetration of F_2_‐Ast@NGs. (A) Fluorescent images of the whole intestinal tract at different time points after oral administration of free Cy5, Ast@NGs, and F_2_‐Ast@NGs. (B) FI curve of the colon and quantitative analysis at 12, 24, and 48 h (*n* = 6). Frozen sections of colitis tissues post (C) 6 h and (D) 48 h administration (Scale bar = 100 µm). (E) 3D CLSM images of free Cy5, Ast@NGs, and F_2_‐Ast@NGs penetrating the FITC‐wheat germ agglutinin‐labeled mucus layer post 48 h administration (Scale bar = 100 µm). Data are presented as mean ± SD and analyzed by Two‐way ANOVA with Tukey's post‐test (B, curve graph on the left) or One‐way ANOVA with Tukey's post‐test (B, bar graph on the right). ns represents not significant, ^*^ *P* < 0.05, ^**^ *P* < 0.01, ^***^ *P* < 0.001, and ^****^ *P* < 0.0001.

### 2.6 Effective Treatment of F_2_‐Ast@NGs Against Colitis Mice

Motivated by the robust environmental resistance, colonic targeting, and mucus penetration, the therapeutic effect of F_2_‐Ast@NGs was evaluated in DSS‐induced colitis mice (Figure 5A). In young mice, fecal occult blood testing indicated serious hematochezia in the Ast group, mild bleeding in the Ast@NGs group, and no visible bleeding in the F_2_‐Ast@NGs group at the treatment endpoint (Figure 5B). Treatment with F_2_‐Ast@NGs produced the greatest therapeutic benefit in young colitis mice, as evidenced by a more pronounced reduction in DAI (Figure 5D) and improved preservation of body weight compared with Ast and Ast@NGs (Figure S14A). In addition, F_2_‐Ast@NG treated mice displayed a marked preservation of colon length relative to the remaining groups, indicating that F_2_‐Ast@NGs could inhibit typical colon shortening induced by DSS in young mice (Figure 5E). In most inflammatory diseases, the extent of inflammation closely correlates with splenomegaly, as more severe inflammation yields a higher spleen‐to‐body‐weight ratio [40]. Accordingly, colitis mice exhibited pronounced splenic enlargement, whereas the ratio in the F_2_‐Ast@NGs group nearly returned to that of the healthy group (Figure S14B). In aged colitis mice, which displayed more severe inflammation, F_2_‐Ast@NGs treatment again prevented hematochezia (Figure 5C), significantly reduced DAI (Figure 5F), and alleviated both body weight loss and splenic enlargement (Figure S14A, B). Colon length results also confirmed the best treatment efficacy of F_2_‐Ast@NGs in aged mice (Figure 5G).

**FIGURE 5:**
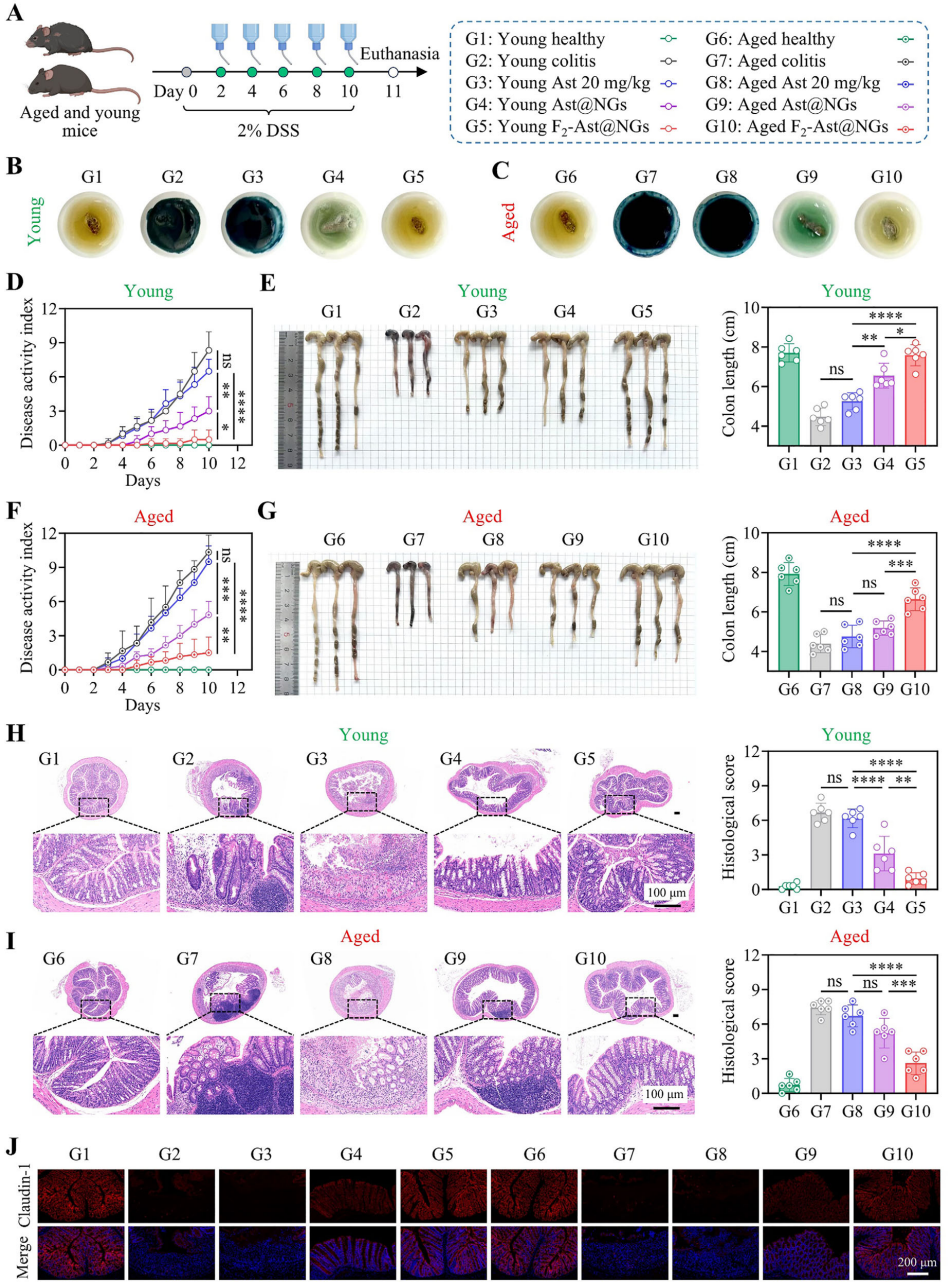
F_2_‐Ast@NGs effectively ameliorate DSS‐induced colitis. (A) Experimental flowchart of NG treatment in colitis mice. Representative fecal samples from (B) young and (C) aged mice following occult blood testing. Curve chart analysis of DAI in (D) young and (F) aged mice (*n* = 6). Representative images of colons and corresponding quantification of colon length in (E) young and (G) aged mice (*n* = 6). Representative H&E‐stained colon sections and quantification of histological scores in (H) young and (I) aged mice (*n* = 6) (Scale bar = 100 µm). (J) Representative immunofluorescence images of claudin‐1 in colon tissues (Scale bar = 200 µm). Data are presented as mean ± SD and analyzed by One‐way ANOVA with Tukey's post‐test (E, G‐I) or Two‐way ANOVA with Tukey's post‐test (D, F). ns represents not significant, ^*^ *P* < 0.05, ^**^ *P* < 0.01, ^***^ *P* < 0.001, and ^****^ *P* < 0.0001.

Histopathological examination of H&E‐stained colon sections from young mice revealed that F_2_‐Ast@NG treatment effectively preserved epithelial architecture, limited inflammatory cell infiltration, and markedly attenuated colonic inflammation (Figure 5H). The histological score decreased from 6.7 in colitis group to 0.9 after F_2_‐Ast@NGs treatment. In parallel, treatment with F_2_‐Ast@NGs effectively restored the expression of the tight junction protein claudin‐1 in the colons of young colitis mice (Figure 5J and Figure S14C). Likewise, in aged colitis mice, F_2_‐Ast@NGs treatment also observably alleviated colonic inflammation, with decreased histological score from 7.4 to 2.6 (Figure 5I), and upregulated claudin‐1 expression by 4.3‐, 3.6‐, and 1.8 fold relative to colitis, Ast, and Ast@NGs groups, respectively (Figure 5J and Figure S14C). Therefore, these results elucidate that F_2_‐Ast@NGs achieve pronounced therapeutic benefits in both young and aged colitis mice, with a more remarkable efficacy in the aged cohort.

To better elucidate the immunomodulatory activity of F_2_‐Ast@NGs, colonic cytokine levels were assessed. In IBD, intestinal epithelial cells and increased immune cells (macrophages, DCs, and CD4^+^ T cells) could secrete different pro‐inflammatory cytokines, such as IL‐6, IL‐1β, TNF‐α, and IL‐17A, to exacerbate inflammation [41, 42]. However, immune regulatory cells, such as T_regs_, could secrete anti‐inflammatory cytokines (IL‐10 and TGF‐β) to counteract this process [43]. Colonic tissues from young colitis mice exhibited significantly increased levels of pro‐inflammatory cytokines, including IL‐6, IL‐1β, TNF‐α, and IL‐17A, compared with healthy controls, and those levels were significantly decreased after F_2_‐Ast@NGs treatment (Figure 6A). In contrast, anti‐inflammatory cytokines IL‐10 and TGF‐β in F_2_‐Ast@NGs groups were significantly increased compared with other groups, implying the activation of regulatory immune cells and restoration of anti‐inflammatory immune responses. In more treatment‐refractory aged colitis mice, F_2_‐Ast@NGs treatment also displayed effective anti‐inflammatory efficacy by decreasing pro‐inflammatory cytokines and increasing anti‐inflammatory cytokines in the colon (Figure 6B). These cytokine results were also supported by the real‐time polymerase chain reaction (real‐time PCR) based analysis of the messenger RNA (mRNA) levels of the selected cytokines. Coincident with the above data, in both young and aged colitis mice, there was a substantial rise in the mRNA levels of IL‐6, IL‐1β, TNF‐α, and IL‐17A, which was rescued by various treatments, with F_2_‐Ast@NGs again outperforming other therapies (Figure S15). In contrast, the mRNA levels of IL‐10 and TGF‐β significantly increased following F_2_‐Ast@NGs treatment.

**FIGURE 6:**
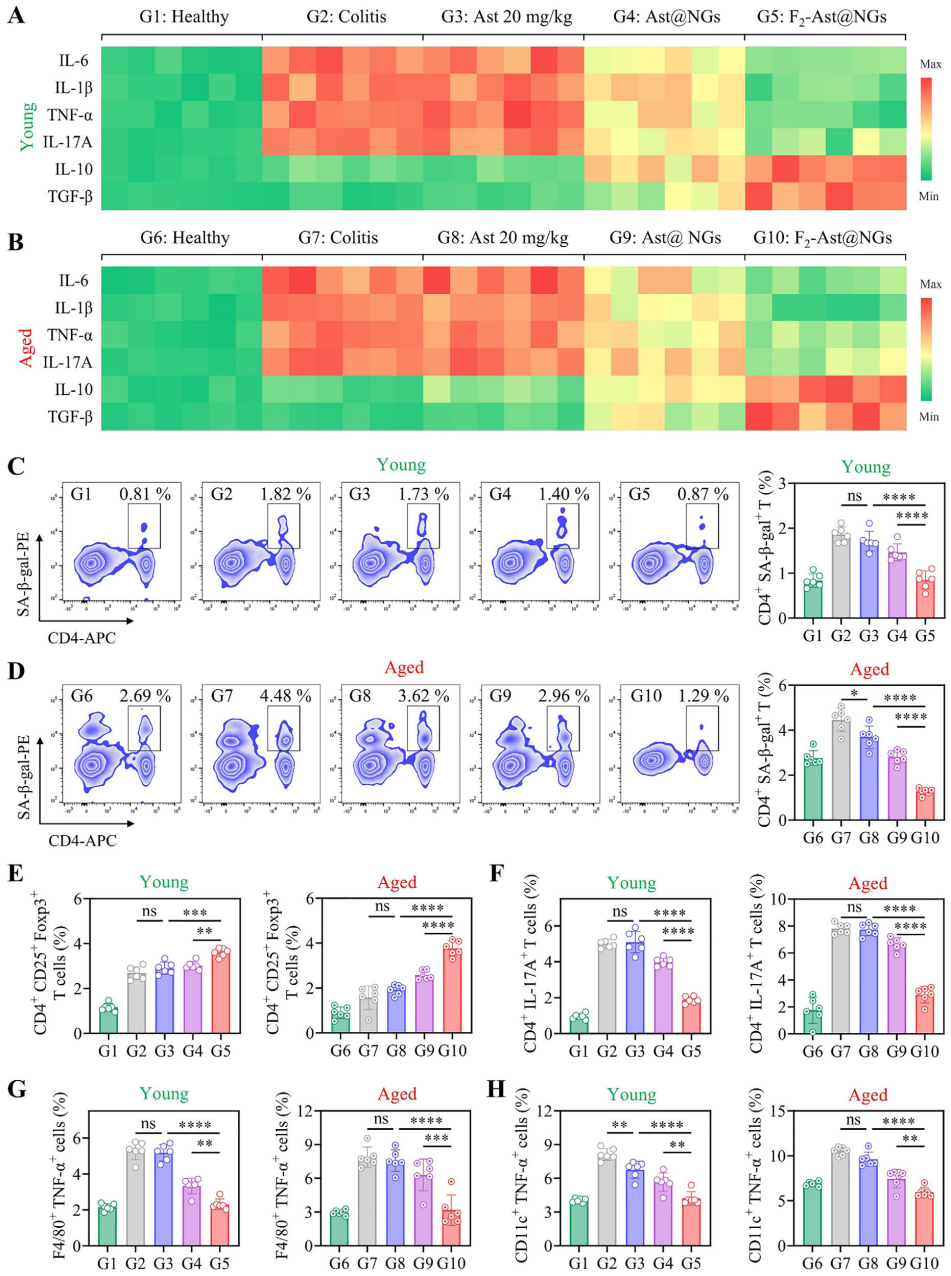
F_2_‐Ast@NGs effectively improve the colonic immune microenvironment. Heatmaps of the relative abundance of pro‐ and anti‐inflammatory cytokines in the colon of (A) young and (B) aged mice (*n* = 6). Representative FC plots and quantitative analysis of CD4^+^ SA‐β‐gal^+^ cells in colonic lamina propria cells from (C) young and (D) aged mice (*n* = 6). (E) Quantitative analysis of CD4^+^ CD25^+^ Foxp3^+^ cells in colonic lamina propria cells (*n* = 6). (F) Quantitative analysis of CD4^+^ IL‐17A^+^ T cells (*n* = 6). (G) Quantitative analysis of TNF‐α^+^ F4/80^+^ cells (*n* = 6). (H) Quantitative analysis of TNF‐α^+^ CD11c^+^ cells (*n* = 6). Data are presented as mean ± SD and analyzed by One‐way ANOVA with Tukey's post‐hoc test. ns represents not significant, ^*^ *P* < 0.05, ^**^ *P* < 0.01, ^***^ *P* < 0.001, and ^****^ *P* < 0.0001.

Colonic immune cell analysis was performed to understand the immune environment. FC analysis showed that the proportion of CD4^+^ PD‐1^+^ T cells in the colon was significantly reduced in young mice after F_2_‐Ast@NGs treatment, displaying 1.5 and 1.3 fold decreases compared with Ast and Ast@NGs treated groups, respectively (Figure S16A). Aged colitis mice exhibited an increased frequency of colonic CD4^+^ PD‐1^+^ T cells, rising from 1.5% in young colitis mice to 3.9% (Figure S16B). F_2_‐Ast@NGs treatment effectively reversed this increase, reducing CD4^+^ PD‐1^+^ T cell frequency by 1.7 and 1.4 fold relative to the Ast and Ast@NGs groups. Moreover, F_2_‐Ast@NGs reduced the proportion of CD4^+^ SA‐β‐gal^+^ T cells by 54.2% in young colitis mice (Figure 6C), and this efficacy was significantly increased in aged colitis mice, reaching 71.5% (Figure 6D). In addition, F_2_‐Ast@NGs decreased P21 expression in CD4 T cells (Figure S17). Younger CD4 T cells possess a higher differentiation potential than aged counterparts [44, 45]. Consistent with the therapeutic effects described in Figure 5, F_2_‐Ast@NGs treatment markedly expanded the CD4^+^ CD25^+^ Foxp3^+^ regulatory T cell population in colonic lamina propria, with 1.3 and 2.4 fold higher compared with young and aged colitis groups, respectively (Figure 6E and Figure S16C, D). IL‐17A is commonly secreted by CD4^+^ T cells, where it exerts potent pro‐inflammatory effects by promoting the recruitment and activation of immune cells [46, 47]. Its frequency was substantially elevated in both young and aged colitis mice but was nearly normalized after F_2_‐Ast@NGs administration (Figure 6F and Figure S16E, F). Furthermore, F_2_‐Ast@NGs treatment markedly reduced the proportion of colonic TNF‐α^+^ F4/80^+^ macrophages and TNF‐α^+^ CD11c^+^ dendritic cells (Figure 6G, H and Figure S18).

These results collectively demonstrate that F_2_‐Ast@NGs effectively attenuate inflammation and restore immune homeostasis by downregulating pro‐inflammatory cytokines, suppressing senescence‐associated CD4^+^ T cells, pathogenic CD4^+^ IL‐17A^+^ T cells, and TNF‐α‐producing myeloid cells, and promoting T_reg_ induction within the inflamed colon.

### 2.7 Regulation of Intestinal SCFAs and Microbiota

SCFAs, including acetate, propionate, butyrate and valerate, play essential roles in maintaining intestinal homeostasis by serving as an energy source for colonocytes, strengthening epithelial barrier integrity and modulating immune responses toward an anti‐inflammatory state [48]. In aged mice, the levels of acetate, propionate, butyrate, and valerate were reduced (Figure 7A and Figure S19), consistent with previous studies [49, 50]. After NGs treatment, SCFA concentrations significantly increased, probably due to microbial fermentation of pectin and β‐glucan within the colon. Notably, the F_2_‐Ast@NGs group exhibited the highest SCFA levels among all groups, which could be attributed to the prolonged colonic retention and sustained local release of the formulation.

**FIGURE 7:**
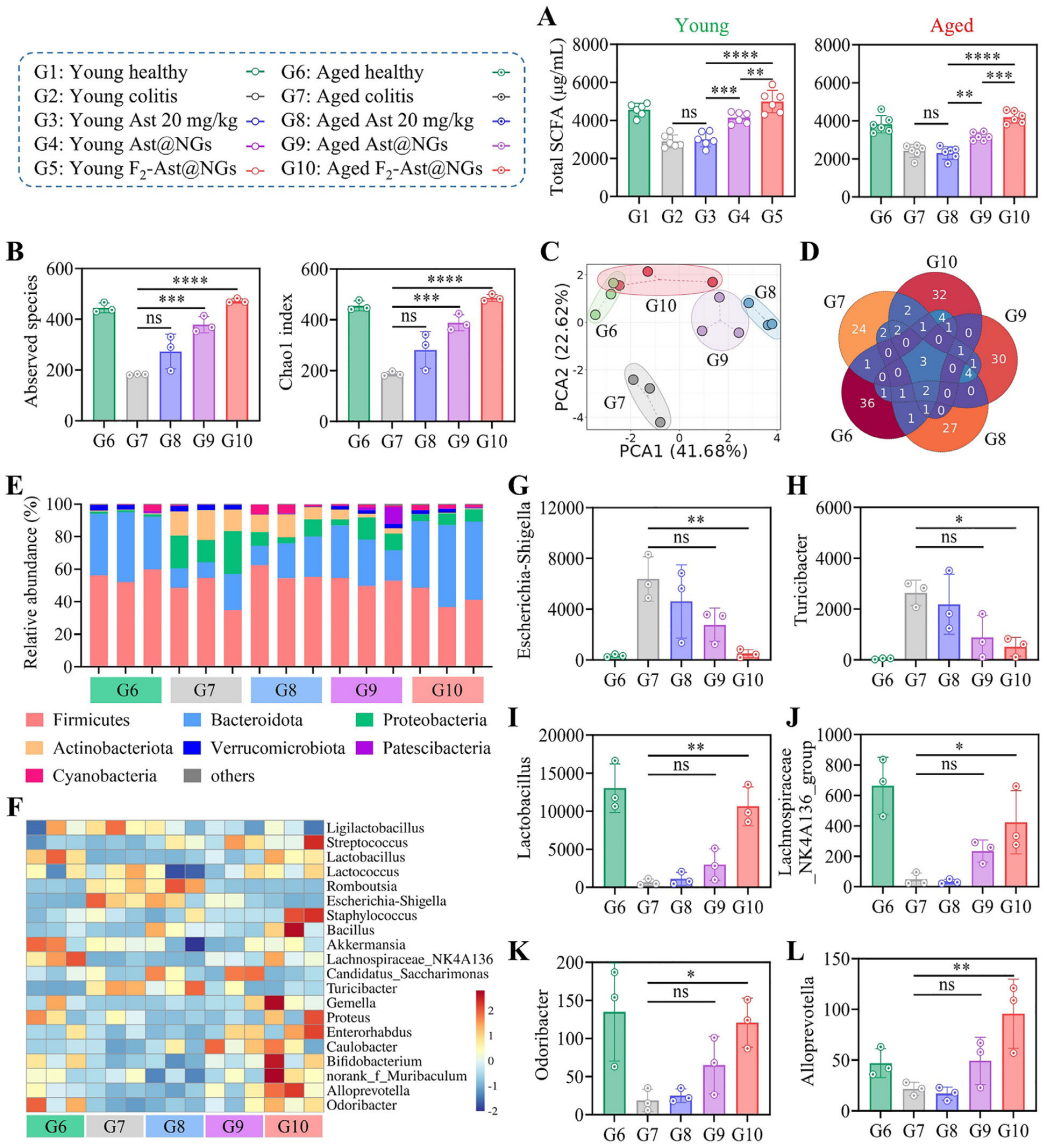
F_2_‐Ast@NGs restored intestinal SCFA levels and intestinal flora homeostasis in DSS‐induced colitis mice. (A) Total intestinal SCFA levels in young and aged mice (*n* = 6). (B) α diversity analysis based on observed species and Chao1 indices (*n* = 3). (C) NMDS ordination plot showing β diversity patterns of the gut microbiota. (D) Venn diagram depicting shared and unique microbial species among different experimental groups (*n* = 3). (E) Relative abundance distribution of gut microbial communities at the phylum level across groups (*n* = 3). (F) Genus‐level heatmap displaying the relative abundance profiles of microbial taxa (rows) from individual mice (*n* = 3). Genus abundance for (G) *Escherichia‐Shigella*, (H) *Turicibacter*, (I) *Lactobacillus*, (J) *Lachnospiraceae_NK4A136_group*, (K) *Odoribacter*, and (L) *Alloprevotella* (*n* = 3). Data are presented as mean ± SD and analyzed by One‐way ANOVA with Tukey's post‐hoc test. ns represents not significant, ^*^ *P* < 0.05, ^**^ *P* < 0.01, ^***^ *P* < 0.001, and ^****^ *P* < 0.0001.

Dysbiosis of gut commensal microbiota is a key contributor to colitis pathogenesis. To determine whether F_2_‐Ast@NGs modulated the gut microbiota, fecal samples collected from aged mice were subjected to 16S rDNA gene amplicon sequencing. Analysis of α diversity revealed that DSS‐induced colitis markedly altered both the richness and diversity of intestinal microbial communities, as reflected by the observed species, Chao1, Shannon, and Simpson indices, compared with healthy, Ast, and Ast@NG groups (Figure 7B and Figure S20). Ordination analysis using non‐metric multidimensional scaling (NMDS) further demonstrated a clear separation of the microbial communities in DSS‐treated mice from those of healthy animals, whereas samples from the F_2_‐Ast@NG group clustered much closer to the healthy microbiota profile (Figure 7C). In addition, a Venn diagram was used to compare the shared and unique microbial taxa among the five experimental groups. Compared with the colitis group, F_2_‐Ast@NGs treatment increased the number of intestinal microbes at the operational taxonomic unit (OTU) level (Figure 7D), reflecting a more diverse microbial community. A more detailed taxonomic analysis of the gut microbiota was subsequently performed at both the phylum and genus levels. The microbial community structure in the F_2_‐Ast@NG group displayed a composition highly comparable to that of the healthy group at the phylum level (Figure 7E). Furthermore, F_2_‐Ast@NG administration markedly enriched *Bacteroidota*, while exerting minimal influence on *Firmicutes* (Figure S21A, B). Importantly, *Proteobacteria*, a phylum strongly associated with inflammation and colitis progression, was markedly suppressed following F_2_‐Ast@NGs treatment (Figure S21C). Analysis of microbial profiles at the genus level showed distinct alterations following F_2_‐Ast@NG administration (Figure 7F). Specifically, treatment with F_2_‐Ast@NGs decreased the abundance of *Escherichia‐Shigella* and *Turicibacter* (Figure 7G, H), two bacterial groups previously associated with intestinal inflammation and colorectal cancer‐related inflammatory conditions [51, 52]. Conversely, F_2_‐Ast@NGs oral administration considerably increased the abundance of *Lactobacillus*, *Lachnospiraceae_NK4A136_group* and *Odoribacter* (Figure 7I–K), the well‐known probiotics that *Lactobacillus* was a genus of lactic acid producing bacteria widely recognized as probiotics that help maintain gut homeostasis and support host health [53], *Lachnospiraceae_NK4A136_group* could produce butyrate and maintain the balance of intestinal microecology [54, 55], and *Odoribacter* is another butyrate producing gut bacterium associated with the preservation of intestinal barrier function and regulation of host immune and metabolic processes [56]. In addition, increased abundance of *Alloprevotella* in the F_2_‐Ast@NGs group was in accordance with our previous results (Figure 7L) [24].

Together, these findings suggest that F_2_‐Ast@NGs treatment is associated with favorable alterations in gut microbial composition, including a reduced abundance of bacterial taxa linked to intestinal inflammation and an increased abundance of beneficial bacteria associated with butyrate production. These changes in SCFA levels and microbial profiles may result from the combined effects of Ast‐mediated immunomodulation and the prebiotic properties of pectin and β‐glucan within the NG matrix, which together contribute to the therapeutic effects against colitis. Given the relatively limited sample size of the microbiome analysis, these findings should be interpreted as supportive evidence of microbiota alterations associated with F_2_‐Ast@NGs treatment. Further validation in larger cohorts and additional functional studies will be required to confirm these observations.

### 2.8 Mechanistic Investigation of F_2_‐Ast@NGs‐Mediated Regulation of CD4^+^ T Cells

To further delineate the contribution of the nanogel matrix to the therapeutic efficacy of F_2_‐Ast@NGs, empty NGs composed of pectin and β‐glucan were prepared and evaluated in aged colitis mice (Figure 8A). Treatment with empty NGs produced a modest improvement in disease severity, as evidenced by a partial reduction in DAI (Figure 8C) and attenuation of body weight loss (Figure S22A). However, NG‐treated mice still exhibited apparent hematochezia (Figure 8B), and although colon length showed a trend toward recovery compared with the colitis group, the difference did not reach statistical significance (Figure 8D). FC analysis further showed that empty NGs did not significantly reduce the proportions of colonic CD4^+^ PD‐1^+^ T cells or CD4^+^ SA‐β‐gal^+^ T cells (Figure 8E and Figure S22B). These findings indicate that the nanogel matrix contributes partially to therapeutic efficacy but is insufficient to reproduce the full immunomodulatory effects of F_2_‐Ast@NGs.

**FIGURE 8:**
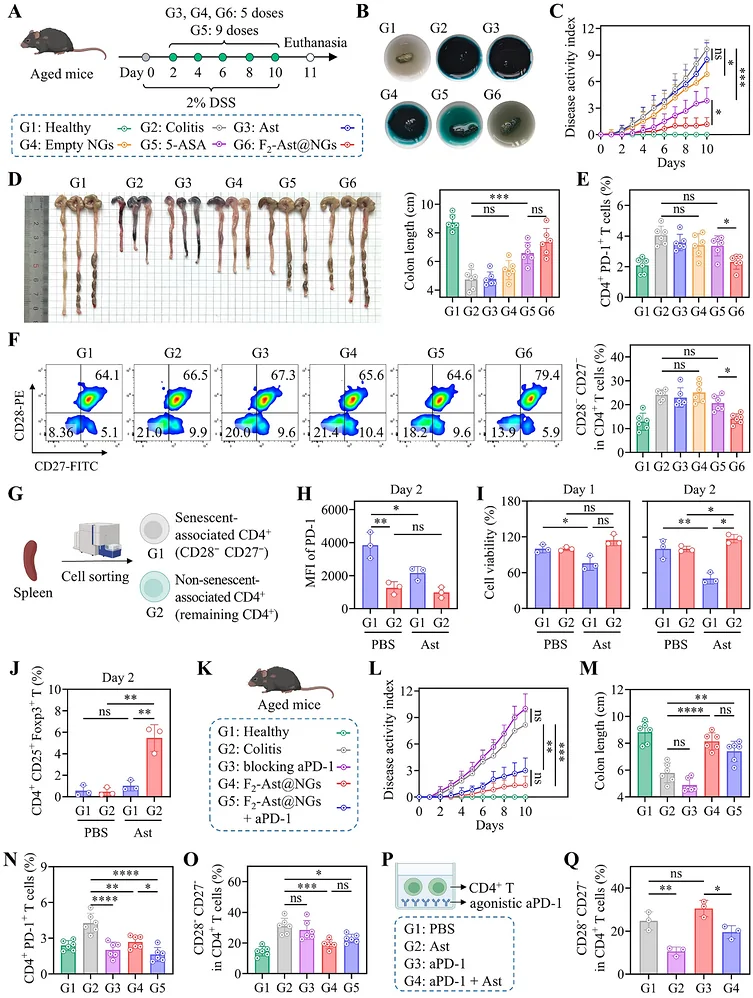
Mechanistic validation of Ast‐mediated regulation of senescence‐associated CD4^+^ T cells in aged colitis. (A) Experimental flowchart of NG treatment in aged colitis mice (*n* = 6). (B) Representative fecal samples following occult blood testing. (C) Curve chart analysis of DAI (*n* = 6). (D) Representative images of colons and corresponding quantification of colon length (*n* = 6). (E) Quantification of PD‐1^+^ CD4 T cells in colonic lamina propria cells (*n* = 6). (F) Representative FC plots and quantitative analysis of CD4^+^ CD27^−^ CD28^−^ T cells in colonic lamina propria cells (*n* = 6). (G) Schematic illustration of the isolation and sorting strategy for senescence‐associated (CD28^−^ CD27^−^) and non‐senescence‐associated (remaining) CD4^+^ T cells. Created with BioRender. (H) MFI of PD‐1 expression on senescence‐associated and non‐senescence‐associated CD4^+^ T cells after treatment with PBS or Ast for 2 days (*n* = 3). (I) Cell viability of senescence‐associated and non‐senescence‐associated CD4^+^ T cells (*n* = 3). (J) Foxp3^+^ T_reg_ differentiation capacity of senescence‐associated and non‐senescence‐associated CD4^+^ T cells in the presence of PBS or Ast for 2 days (*n* = 3). (K) Experimental groups for therapeutic evaluation in aged colitis mice. (L) Curve chart analysis of DAI (*n* = 6). (M) Quantification of colon length (*n* = 6). Quantitative analysis of (N) CD4^+^ PD‐1^+^ T cells and (O) CD4^+^ CD27^−^ CD28^−^ T cells in colonic lamina propria cells (*n* = 6). (P) Schematic illustration of CD4^+^ T cells cultured in the presence of plate‐bound agonistic aPD‐1 or Ast. Created with BioRender. (Q) Quantitative analysis of CD4^+^ CD27^−^ CD28^−^ T cells following treatment with plate‐bound agonistic aPD‐1 or Ast (*n* = 3). Data are presented as mean ± SD and analyzed by Two‐way ANOVA with Tukey's post‐test (C, L) or One‐way ANOVA with Tukey's post‐test (D‐F, H‐J, M‐O, Q). ns represents not significant, ^*^ *P* < 0.05, ^**^ *P* < 0.01, ^***^ *P* < 0.001, and ^****^ *P* < 0.0001.

We next compared F_2_‐Ast@NGs with 5‐aminosalicylic acid (5‐ASA), a clinically approved drug for IBD [1]. Treatment with 5‐ASA significantly alleviated colitis symptoms, as evidenced by attenuated body weight loss, decreased fecal bleeding, reduced DAI, and improved colon length (Figure 8B–D and Figure S22A). Under the tested conditions, F_2_‐Ast@NGs produced greater therapeutic benefits than 5‐ASA. Furthermore, FC analysis revealed that 5‐ASA treatment did not significantly alter the proportions of colonic CD4^+^ PD‐1^+^ or CD4^+^ SA‐β‐gal^+^ T cells, whereas F_2_‐Ast@NGs significantly reduced both populations (Figure 8E and Figure S22B). These results highlight additional regulatory effects of F_2_‐Ast@NGs on senescence‐associated CD4^+^ T cell phenotypes that were not observed with 5‐ASA under the present conditions.

Given that loss of the co‐stimulatory receptors CD27 and CD28 is a well‐recognized phenotypic feature associated with T cell senescence [14], we incorporated the CD27^−^ CD28^−^ phenotype as an additional indicator complementary to SA‐β‐gal activity and P21 expression. Consistent with the reduction in CD4^+^ SA‐β‐gal^+^ T cells (Figure S22B), F_2_‐Ast@NGs treatment significantly decreased the proportion of CD27^−^ CD28^−^ CD4^+^ T cells in the colonic lamina propria compared with colitis mice, whereas neither 5‐ASA nor empty NGs produced a significant effect (Figure 8F). Together, these findings provide additional evidence that F_2_‐Ast@NGs attenuate senescence‐associated features of colonic CD4^+^ T cells.

To further clarify the differential effects of Ast on distinct CD4^+^ T cell populations, CD4^+^ CD27^−^ CD28^−^ T cells were isolated from the spleens of aged mice by FC sorting and defined as senescence‐associated CD4^+^ T cells, whereas the remaining CD4^+^ T cells were classified as non‐senescence‐associated CD4^+^ T cells (Figure 8G). Senescence‐associated CD4^+^ T cells exhibited markedly higher PD‐1 expression than non‐senescence‐associated CD4^+^ T cells, and Ast treatment significantly reduced PD‐1 expression in this population (Figure 8H). Cell viability analysis further showed that Ast preferentially reduced the metabolic activity of senescence‐associated CD4^+^ T cells while exerting minimal effects on non‐senescence‐associated CD4^+^ T cells (Figure 8I). Moreover, senescence‐associated CD4^+^ T cells exhibited a limited capacity to differentiate into CD25^+^ Foxp3^+^ T_regs_, whereas Ast treatment markedly enhanced Foxp3^+^ T_reg_ differentiation from non‐senescence‐associated CD4^+^ T cells, with an approximately five fold increase compared with Ast‐treated senescence‐associated CD4^+^ T cells (Figure 8J). In parallel, Ast significantly reduced IL‐17A production by senescence‐associated CD4^+^ T cells (Figure S23). Collectively, these findings suggest that Ast differentially regulates these CD4^+^ T cell populations by preferentially suppressing the metabolic activity and pro‐inflammatory phenotype of senescence‐associated CD4^+^ T cells while enhancing the T_reg_ differentiation potential of non‐senescence‐associated CD4^+^ T cells.

Given the elevated PD‐1 expression observed in senescence‐associated CD4^+^ T cells, we next investigated whether modulation of PD‐1 signaling could account for the effects of Ast. Aged DSS‐induced colitis mice were treated with a blocking anti‐PD‐1 antibody (aPD‐1), F_2_‐Ast@NGs, or their combination (Figure 8K). Interestingly, aPD‐1 monotherapy did not alleviate colitis. Instead, aPD‐1‐treated mice exhibited greater disease severity than untreated colitis mice, as evidenced by increased DAI (Figure 8L) and reduced colon length (Figure 8M and Figure S24). Moreover, combination treatment with aPD‐1 partially attenuated the therapeutic benefits of F_2_‐Ast@NGs alone (Figure 8L, M and Figure S24). FC analysis showed a lower detectable proportion of CD4^+^ PD‐1^+^ T cells following aPD‐1 treatment (Figure 8N), whereas the proportion of CD4^+^ CD27^−^ CD28^−^ senescence‐associated T cells remained unchanged (Figure 8O). Notably, the apparent reduction in detectable CD4^+^ PD‐1^+^ T cells may partly reflect epitope masking caused by occupancy of PD‐1 by the blocking antibody, thereby interfering with subsequent FC detection. To further examine whether enhanced PD‐1 signaling could interfere with the effects of Ast, splenic CD4^+^ T cells isolated from aged mice were treated with an agonistic aPD‐1 antibody. Ast still significantly reduced the proportion of CD4^+^ CD27^−^ CD28^−^ senescence‐associated T cells despite PD‐1 activation (Figure 8P, Q). Collectively, these findings indicate that modulation of PD‐1 signaling alone is insufficient to account for the effects of Ast on senescence‐associated CD4^+^ T cell phenotypes, suggesting that Ast acts through mechanisms extending beyond direct regulation of the PD‐1 pathway.

Overall, these findings indicate that the therapeutic activity of F_2_‐Ast@NGs reflects both the contribution of the pectin/β‐glucan nanogel matrix and the efficient colonic delivery of Ast. Ast differentially regulates senescence‐associated and non‐senescence‐associated CD4^+^ T cell populations, while its effects cannot be explained solely by modulation of PD‐1 signaling. Further studies are required to define the broader molecular pathways involved.

### 2.9 Essential Role of Foxp3^+^ T_regs_ in F_2_‐Ast@NGs‐Mediated Therapeutic Efficacy

To elucidate the role of Foxp3^+^ T_regs_ in the immunomodulatory mechanism of F_2_‐Ast@NGs, a DSS‐induced colitis model was established in Foxp3^+^ T_reg_‐deficient mice (Figure 9A). Compared with wild‐type colitis mice, Foxp3^+^ T_reg_‐deficient colitis mice display more severe conditions with low body weight (Figure S25) and high DAI (Figure 9B), and the length of the colon was further shortened from 5.5 to 4.3 cm (Figure 9C), highlighting the indispensable role of T_regs_ in suppressing intestinal inflammation. Following F_2_‐Ast@NGs treatment, T_reg_‐deficient mice exhibited markedly attenuated therapeutic responses compared with wild‐type colitis mice (Figure 9B, C). The effective therapeutic efficacy of F_2_‐Ast@NGs was also observed in wild‐type colitis mice when compared with Foxp3^+^ T_reg_‐deficient colitis mice. Histopathological analysis further revealed that colon tissues from T_reg_‐deficient mice treated with F_2_‐Ast@NGs exhibited extensive epithelial damage, crypt abnormalities, goblet cell loss, and pronounced inflammatory infiltration in the lamina propria, whereas wild‐type colitis mice displayed largely preserved colonic morphology following treatment (Figure 9D). These findings collectively indicated that the therapeutic efficacy of F_2_‐Ast@NGs relies predominantly on Foxp3^+^ T_reg_‐mediated immunoregulation, which is essential for preserving intestinal barrier function and limiting colitis progression.

**FIGURE 9:**
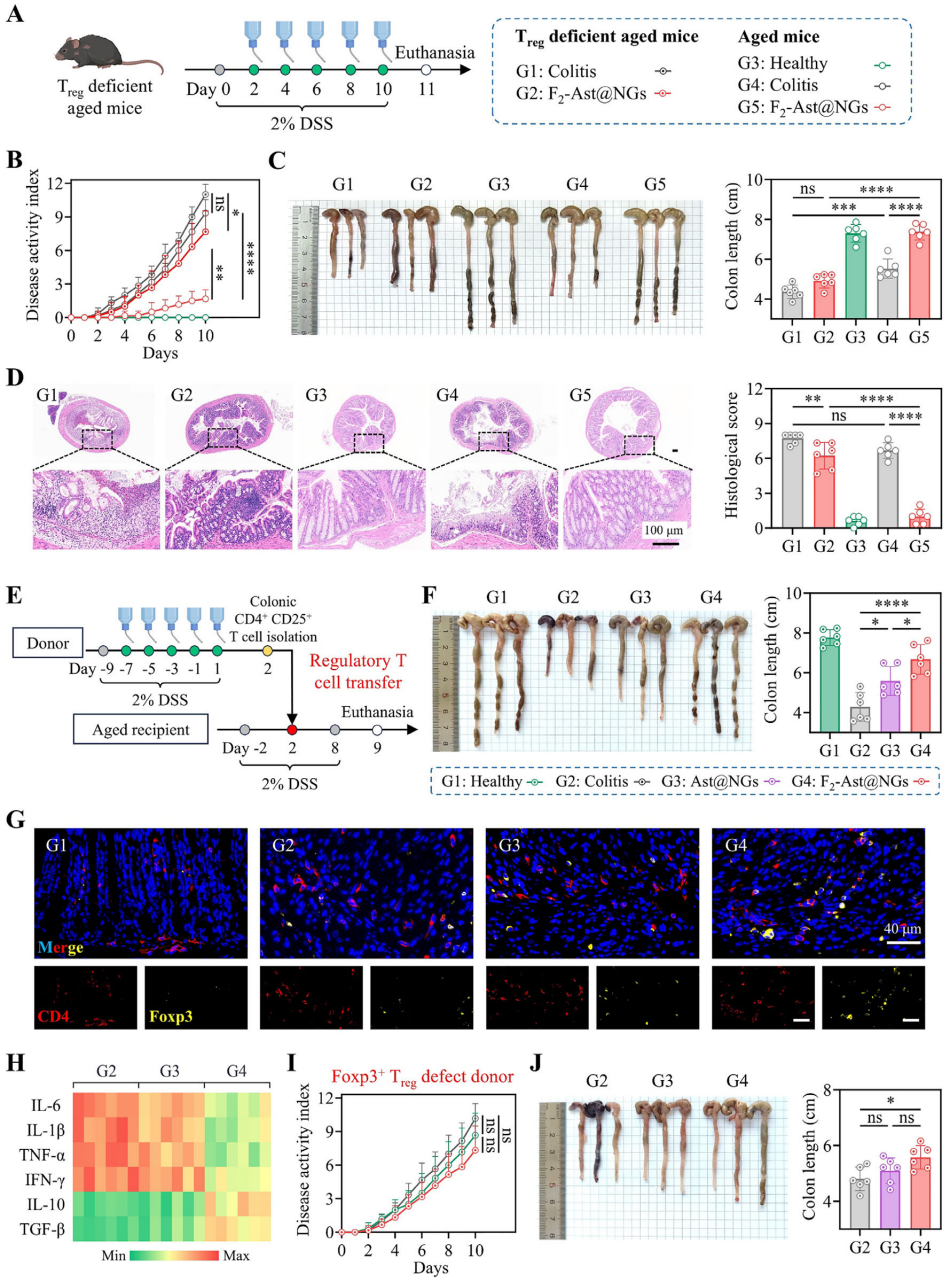
Abolished therapeutic efficacy of F_2_‐Ast@NGs in T_reg_‐deficient colitis mice. (A) Experimental flowchart of F_2_‐Ast@NGs treatment of DSS‐induced T_reg_‐deficient colitis mice. (B) Curve chart analysis of DAI (*n* = 6). (C) Representative images of colons and corresponding quantification of colon length (*n* = 6). (D) Representative H&E stained colon sections and quantification of histological scores (*n* = 6) (Scale bar = 100 µm). (E) Experimental flowchart of regulatory T cell transfer for the treatment of DSS‐induced aged colitis mice. (F) Representative images of colons and corresponding quantification of colon length (*n* = 6). (G) Immunofluorescent staining images of the colon. Scale bars, 40 µm. Blue, DAPI; Red, CD4; Yellow, Foxp3. (H) Heatmaps of relative abundance of pro‐ and anti‐inflammatory cytokines in the colon of mice (*n* = 6). (I) Curve chart analysis of DAI in Foxp3^+^ T_reg_‐deficient donor mice (*n* = 6). (J) Representative images of colons in Foxp3^+^ T_reg_‐deficient donor mice and corresponding quantification of colon length (*n* = 6). Data are presented as mean ± SD and analyzed by Two‐way ANOVA with Tukey's post‐test (B, I) or One‐way ANOVA with Tukey's post‐test (C, D, F, J). ns represents not significant, *P < 0.05, ^**^ *P* < 0.01, ^***^ *P* < 0.001, and ^****^ *P* < 0.0001.

To directly determine whether T_regs_ elicited by F_2_‐Ast@NGs mediate its therapeutic benefit, we conducted adoptive T_reg_ transfer experiments in UC mice (Figure 9E). CD4^+^ CD25^+^ T cells (enriched for T_regs_) were isolated from colonic tissue of mice treated with Ast@NGs or F_2_‐Ast@NGs. Cells were sorted using surface CD4 and CD25 only, without Foxp3 selection, because Foxp3 staining requires membrane permeabilization, which reduces viability and prevents successful transfer. Using wild‐type donors, transfer of CD4^+^ CD25^+^ T cells from F_2_‐Ast@NGs‐treated mice markedly mitigated DAI score (Figure S26) and improved colon shortening (Figure 9F) compared with the Ast@NGs‐treated group. Moreover, recipients also showed an increased colonic T_reg_ frequency (Figure 9G), accompanied by remodeling of the local immune milieu (Figure 9H). To specifically probe the contribution of Foxp3^+^ T_regs_ induced by F_2_‐Ast@NGs, we next used Foxp3^+^ T_reg_ deficient mice as donors. In this setting, CD4^+^ CD25^+^ cells obtained after F_2_‐Ast@NG treatment conferred substantially reduced protection, yielding outcomes comparable to the Ast@NGs group (Figure 9I, J). Collectively, these results indicate that F_2_‐Ast@NGs drive a Foxp3^+^ T_reg_‐dependent immunoregulatory program that is essential for their therapeutic efficacy.

### 2.10 Biosafety Evaluation of NGs

For biosafety evaluation, a hemolysis assay was performed initially. A negligible hemolysis (< 7%) was observed after incubation with Ast@NGs and F_2_‐Ast@NGs (Figure 10A), indicating good hemocompatibility and suitability for further biological applications. Subsequently, aged C57BL/6 mice were consecutively administered Ast, Ast@NGs, or F_2_‐Ast@NGs every two days for a total of 5 doses (Figure 10B). Serum biochemical analysis showed comparable levels of alanine aminotransferase (ALT) and aspartate aminotransferase (AST) among all groups (Figure S27A), indicating preserved hepatic function. Similarly, uric acid (UA) and creatinine (CR) levels were comparable to those in the healthy group (Figure S27B), suggesting unimpaired renal function. Hematological parameters remained within normal physiological ranges (Figure S27C). Moreover, histological examination of intestinal segments (duodenum, jejunum, ileum, colon) revealed no evidence of structural damage or inflammation (Figure 10C). Consistently, major organs (heart, liver, spleen, lung, kidney) displayed normal morphology after treatment with Ast@NGs or F_2_‐Ast@NGs (Figure 10D).

**FIGURE 10:**
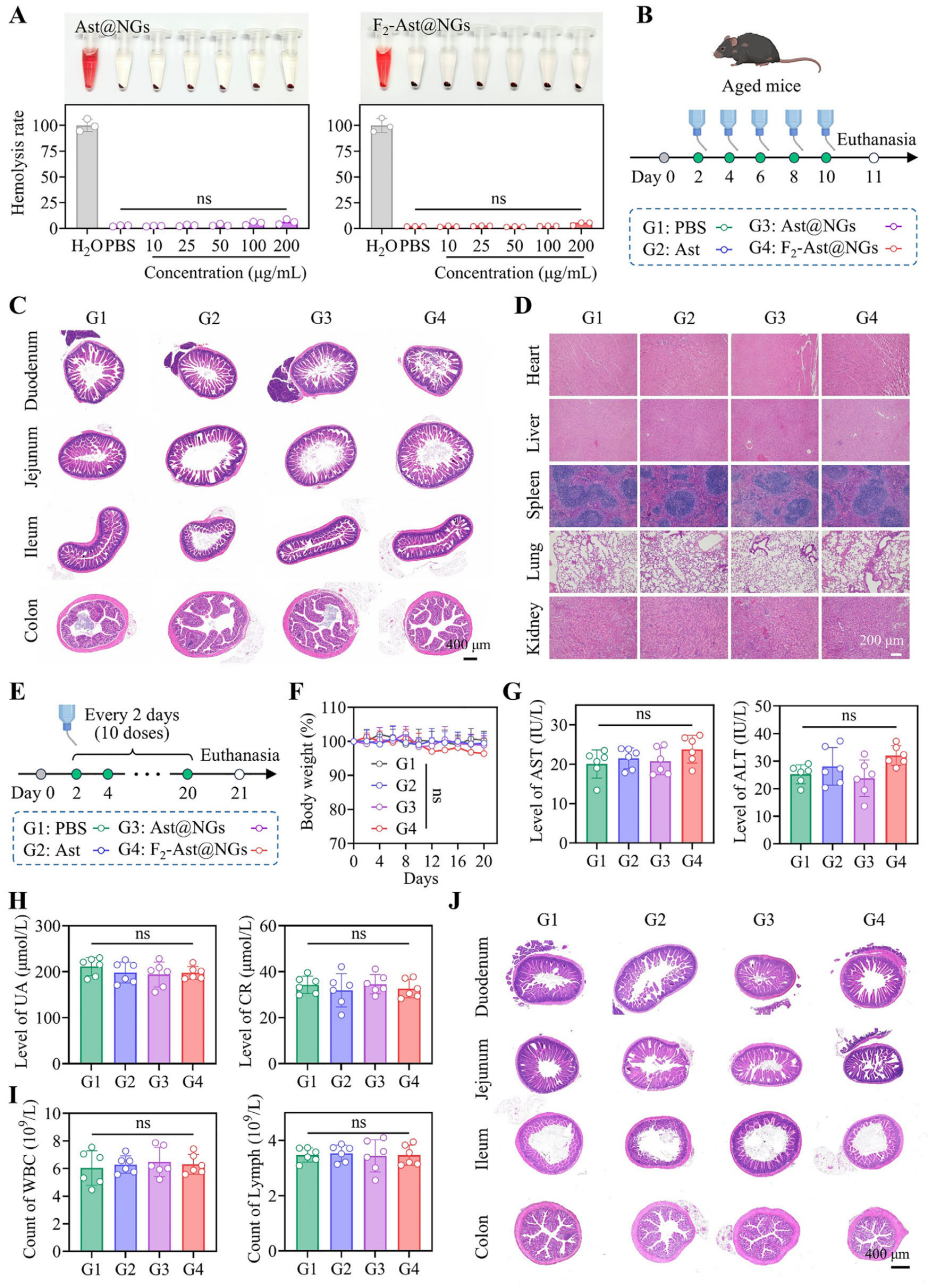
Biosafety evaluation of Ast@NGs and F_2_‐Ast@NGs in vitro and in vivo. (A) Hemolysis test with different concentrations of Ast@NGs and F_2_‐Ast@NGs (*n* = 3). (B) Experimental procedure for biosafety evaluation in aged C57BL/6 mice (*n* = 6). (C) Representative H&E‐stained sections of duodenum, jejunum, ileum, and colon. Scale bar, 400 µm. (D) Representative H&E‐stained sections of heart, liver, spleen, lung, and kidney. Scale bar, 200 µm. (E) Experimental procedure for the three‐week biosafety evaluation in aged C57BL/6 mice (*n* = 6). (F) Curve chart variations of murine body weight across time (*n* = 6). (G) The levels of alanine aminotransferase (ALT) and aspartate aminotransferase (AST) in serum (*n* = 6). (H) The levels of uric acid (UA) and creatinine (CR) in serum (*n* = 6). (I) The counts of white blood cells (WBC) and lymphocytes (Lymph) (*n* = 6). (J) Representative H&E‐stained sections of duodenum, jejunum, ileum and colon. Scale bar, 400 µm. Data are presented as mean ± SD and analyzed by One‐way ANOVA with Tukey's post‐test (A, G‐I) or two‐way ANOVA with Tukey's post‐test (F). ns represents not significant.

To further evaluate the safety of repeated administration, an additional three‐week study was performed in healthy aged mice. Ast, Ast@NGs, or F_2_‐Ast@NGs were orally administered every two days for a total of 10 doses (Figure 10E). No significant body weight loss was observed throughout the treatment period (Figure 10F). At the end of the study, serum biochemical and hematological parameters showed no significant abnormalities (Figure 10G–I and Figure S28), and H&E staining revealed no apparent histopathological alterations in the duodenum, jejunum, ileum, or colon (Figure 10J).

Collectively, these results demonstrate that F_2_‐Ast@NGs exhibited a favorable biosafety profile following both short‐term and repeated administration under the tested conditions. Despite the favorable safety observed after repeated administration, the long‐term safety of the PFOA‐containing nanogel formulation requires further evaluation because of concerns regarding PFOA persistence, bioaccumulation, and chronic toxicity. Future studies should therefore characterize its long‐term biodistribution, metabolism, accumulation, and elimination. PEGylation may provide an alternative strategy for reducing mucin interactions, although repeated exposure can induce anti‐PEG immune responses [57, 58]. Direct comparison of fluorinated and PEGylated formulations will therefore be valuable for evaluating their relative mucus‐penetrating performance and long‐term safety. Given the chronic nature of IBD, longer repeated‐dose studies will be needed before an appropriate clinical treatment duration can be defined.

## 3 Conclusions

In summary, we successfully developed a fluorinated NG formulation (F_2_‐Ast@NGs) that effectively overcomes the GI instability of Ast and achieves targeted drug release in the inflamed colon. The β‐glucan shell provided robust protection against acidic and bile salt degradation while allowing β‐glucanase‐responsive disassembly in the colon. Meanwhile, the amphiphobic fluorocarbon segments facilitated efficient mucus penetration, and the mucoadhesive pectin component prolonged colonic retention, ensuring sustained drug accumulation at inflamed sites.

Mechanistically, Ast differentially regulates distinct CD4^+^ T cell populations, preferentially suppressing the metabolic activity and pro‐inflammatory phenotype of senescence‐associated CD4^+^ T cells while promoting T_reg_ differentiation from non‐senescence‐associated CD4^+^ T cells. These effects contribute to the restoration of immune homeostasis and attenuation of intestinal inflammation. In parallel, the β‐glucan/pectin nanogel matrix contributes to increased SCFA production and favorable alterations in gut microbial composition, providing complementary benefits to the overall therapeutic efficacy.

Altogether, our findings highlight a multifunctional NG platform that integrates GI protection, mucus penetration, microbiota modulation, and local immune modulation to achieve precise and sustained therapy for colitis in aged hosts. Beyond demonstrating therapeutic efficacy, this work provides a broadly applicable framework for designing microbiota‐responsive and immunomodulatory oral nanomedicines for chronic inflammatory disorders. Future studies are warranted to further elucidate the molecular mechanisms underlying Ast‐mediated regulation of CD4^+^ T cell populations and to evaluate the long‐term biosafety and durability of therapeutic responses.

## 4 Materials and Methods

### 4.1 Materials

Astilbin (purity > 98.0%) was extracted from *Smilax glabra Roxb*. by our laboratory. High‐methoxylated pectin (from citrus peel, galacturonic acid ≥ 74.0%), β‐D‐glucan (from barley), PEG (MW 4 kDa) and β‐1,3‑D‐glucanase (from *Helix pomatia*) were purchased from Sigma‐Aldrich (USA). 5‐aminosalicylic acid (5‐ASA), sodium chloride (NaCl), zinc chloride (ZnCl_2_), and perfluorooctanoic acid (PFOA) were obtained from Aladdin (China). Dextran sodium sulfate (DSS) was obtained from Seebio (China). Fecal occult blood test kit and 4’,6‐diamidino‐2‐phenylindole (DAPI) were purchased from Shanghai Yuanye Bio‐Technology Co., Ltd (China). HBSS and HEPES were obtained from Solarbio (China). β‑glucanase activity testing kit was obtained from Mlbio (China). Live/Dead Fixable Dead Cell Stain Kits, CellTrace CFSE, and IL‐6, IL‐1β, TNF‐α, IL‐17A, IL‐10, and TGF‐β ELISA kits were obtained from Thermo Fisher (USA). RPMI 1640, fetal bovine serum (FBS), and fetal calf serum (FCS) were obtained from Gibco (USA). EasySep Mouse CD4^+^ T Cell Isolation Kit plus the EasySep Magnet were purchased from STEMCELL (Canada). Cell Meter Cellular Senescence Activity Assay Kit Red Fluorescence was purchased from Ruixi Bio (China). Type IV collagenase, DNaseI, dispase, FITC‐labeled wheat germ agglutinin, acetic acid, propionic acid, butyric acid, valeric acid, and diphtheria toxin were obtained from Sigma‐Aldrich. Fecal Genome DNA Extraction Kit was purchased from BioTek (China). Alamar Blue kit was obtained from Yeasen (China).

Murine antibodies of CD16/32 (Clone 93), CD4‐APC (Clone GK1.5), PD‐1‐APC‐Cy7 (Clone 29F.1A12), CD25‐PE (Clone 3C7), CD45‐FITC (Clone 30‐F11), IL‐17A‐APC‐Cy7 (Clone TC11‐18H10.1), F4/80‐FITC (Clone BM8), CD11c‐APC (Clone N418), TNF‐α‐PE (Clone MP6‐XT22), CD28‐PE (Clone 37.51) and CD27‐FITC (Clone LG.3A10) for flow cytometric (FC) analysis were purchased from Biolegend (USA). Murine antibodies of Foxp3‐PE‐Cy7 (Clone 236A/E7) for FC analysis were obtained from Invitrogen (USA). Murine antibodies of P21 (Clone EPR18021) were purchased from Abcam (USA). Purified blocking anti‐mouse PD‐1 (Clone RMP1‐14), agonistic anti‐mouse PD‐1 (Clone RMP1‐30), anti‐mouse CD3 (Clone 17A2), and anti‐mouse CD28 (Clone 37.51) were purchased from BioXcell (USA).

### 4.2 Mice and Colitis Model

Young (2–3 months old) and aged (20–24 months old) female C57BL/6 mice were purchased from Cavens Experimental Animal Technology (China). C57BL/6 Smoc‐Foxp3^em3(IRES‐DTR)Smoc^ (Foxp3‐IRES‐DTR) mice were obtained from Shanghai Model Organisms Center (China). Animal studies were performed following the Guide for the Care and Use of Laboratory Animals, with all protocols approved by the Animal Care and Welfare Committee of Soochow University (SYXK [Su] 2021‐0065).

Experimental colitis was established by providing mice with 2% (w/v) DSS in drinking water ad libitum. Body weight and disease activity index (DAI) were monitored each day. The DAI was determined based on the combined scores for body weight loss (0: < 1%; 1: 1%–5%; 2: 5%–10%; 3: 10%–15%; 4: > 15%), stool consistency (0: normal; 2: loose stool; 4: watery diarrhea), and fecal bleeding (0: normal; 2: slight bleeding; 4: severe bleeding), each scored independently. Fresh fecal samples were collected on the last day, and gastrointestinal bleeding was evaluated using a fecal occult blood test kit. After euthanasia via carbon dioxide (CO_2_) inhalation, colons were excised, placed on grid paper, and photographed.

Colon samples were embedded in optimal cutting temperature compound and maintained at ‐80°C overnight. Frozen sections (10 µm) were subsequently prepared and subjected to H&E staining for histopathological evaluation. Pathological scores were assigned independently by three blinded researchers based on intestinal epithelial morphology (0, normal; 1, partial loss of goblet cells; 2, massive loss of goblet cells; 3, partial loss of crypts; 4, severe loss of crypts) and inflammatory infiltration (0, normal; 1, inflammatory cells infiltrate the crypts; 2, inflammatory cells infiltrate the muscular layer of the mucosa; 3, large numbers of inflammatory cells infiltrate the muscular layer of the mucosa; 4, inflammatory cells infiltrate the submucosa).

### 4.3 FC Analysis of Colonic Immune Cells and Cytokine Analysis

Colons were minced and incubated at 37 °C in HBSS containing 1% HEPES to remove epithelial cells, followed by three washes with stirring. Residual tissue was digested in HBSS supplemented with 10% FCS (v/v), type IV collagenase (1  mg mL^−1^), DNase I (0.5  mg mL^−1^) and dispase (0.5  mg mL^−1^) at 37 °C for 1 h with continuous magnetic stirring. The digested tissues were passed through a 70 µm cell strainer, and the collected cells were washed with PBS before further analysis.

The isolated colonic lamina propria cells were suspended in PBS containing 2% FBS (v/v). Cells were stained with Live/Dead reagent at room temperature (RT) for 20 min to distinguish live cells, followed by incubation with CD16/32 antibody at RT for 10 min to block surface Fc receptors. Subsequently, cells were stained with various surface fluorescently conjugated antibodies at RT for 20 min. After that, all cells were fixed with cell fixation solution at RT for 30 min for subsequent FC analysis. For intracellular staining, after finishing the staining of cell surface markers, cells were incubated with cell permeabilization buffer at RT for 20 min and then stained with different intracellular FC antibodies at RT for 20 min. Prior to FC analysis, all samples were passed through a 200‐mesh nylon filter. Because several antibodies used in this study shared the same fluorophores, different readouts were analyzed in separate staining panels rather than in a single multicolor panel.

For colonic cytokines, collected colons were homogenized in RIPA buffer on ice at 5000 rpm for 30 s, lysed at 4°C for 2 h, and centrifuged at 12 000 × g for 20 min at 4°C. Supernatants were analyzed using ELISA kits.

### 4.4 Bioinformatics Analysis

The association between *PDCD1* expression, which encodes PD‐1, and age at diagnosis in patients with colitis was analyzed using publicly available datasets from Gene Expression Omnibus. A total of 106 eligible patients were included in the final analysis. The correlation between *PDCD1* expression and age at diagnosis was evaluated using Pearson correlation analysis. Patients were further stratified into younger (< 60 years) and aged (≥ 60 years) groups according to the commonly accepted definition of elderly‐onset IBD.

### 4.5 Comparison Between Young and Aged Colitis Model

Aged and young C57BL/6 mice were randomly divided into 4 groups (*n* = 6): Young healthy, Aged healthy, Young colitis, and Aged colitis. 2% DSS water was supplied from day 0 to 10. Mice were euthanized on day 11. Colons were collected for length measurement and H&E staining. Colonic lamina propria cells were stained with Live/Dead solution, anti‐mouse CD45, anti‐mouse CD4, anti‐mouse PD‐1, SA‐β‐gal, anti‐mouse P21, anti‐mouse CD25, and anti‐mouse Foxp3 for FC analysis.

### 4.6 Colitogenic CD4^+^ T Cell Transfer Experiment

Aged and young C57BL/6 mice were supplied with 2% DSS from day 0 to 10 to induce colitis. CD4^+^ T cells were subsequently isolated from colonic lamina propria via a CD4^+^ T Cell Isolation Kit according to the manufacturer's protocol. Recipient young mice, which also received consecutive 2% DSS water from day 0 to 8, received an *i.v*. injection of 1 × 10^6^ colitogenic CD4^+^ T cells on day 2. All mice were sacrificed on day 9, and colons were excised for length measurement, followed by the determination of IL‐17A levels in the colon.

### 4.7 Cell Isolation for In Vitro Culture and Analysis

The collected colonic lamina propria cells were suspended in RPMI 1640 medium containing 10% (v/v) FBS, 2% (v/v) penicillin‐streptomycin solution, and 100 IU mL^−1^ IL‐2 for culture in 6‐well plates. After 24 h, anti‐CD3 (0.5 µg mL^−1^) and anti‐CD28 (0.5 µg mL^−1^) antibodies were added to each well, and Ast was added to the required wells at different final concentrations (10, 50, 100, 250, 500, and 1000 ng mL^−1^). After another 24 h of incubation, cells were collected and stained using Live/Dead solution, anti‐mouse CD45, anti‐mouse CD4, anti‐mouse PD‐1, and anti‐mouse P21 for FC analysis.

To assess the functional status of Ast‐treated senescent CD4^+^ T cells, their proliferative capacity and differentiation potential were evaluated. Splenocytes were harvested from aged colitis mice, followed by erythrocyte depletion using ACK lysis buffer. The remaining cells were labeled with CFSE and then cultured in plates pre‐coated with anti‐CD3 and anti‐CD28 antibodies in medium supplemented with 100 IU mL^−1^ IL‐2 with or without Ast (500 ng mL^−1^). After 2 days of incubation, the cells were harvested and stained with Live/Dead dye, anti‐mouse CD4, anti‐mouse CD25, and anti‐mouse Foxp3 for FC analysis.

### 4.8 Therapeutic Efficacy of Free Ast in Colitis Mice

Aged and young C57BL/6 mice were randomly divided into 6 groups (*n* = 6): Young colitis, Young 20 mg kg^−1^ Ast, Young 100 mg kg^−1^ Ast, Aged colitis, Aged 20 mg kg^−1^ Ast, Aged 100 mg kg^−1^ Ast. All groups received 2% DSS from day 0 to day 10. Ast was suspended in 0.5 % CMC‐Na and administered by oral gavage every 2 days from day 2 to 10, with five doses in total. All mice were sacrificed on day 11, and colons were collected for length measurement. Colonic lamina propria cells were stained with Live/Dead solution, anti‐mouse CD45, anti‐mouse CD4, anti‐mouse PD‐1, SA‐β‐gal, anti‐mouse CD25, and anti‐mouse Foxp3 for FC analysis.

### 4.9 RNA Sequencing and Differential Gene Enrichment Analysis

Collected colon tissues were preserved in nitrogen immediately. Total RNA was isolated according to the manufacturer's instructions. RNA libraries were subsequently constructed and quantified based on their effective library concentrations. Sequencing was performed on the MGISEQ‐T7 platform, and transcript‐level quantification was conducted using HTSeq. Subsequent bioinformatic analyses, including differential expression and enrichment analyses, were carried out using the online platform https://www.bioinformatics.com.cn/.

### 4.10 Synthesis of Nanogels (NGs)

Pectin (10 mg mL^−1^) was dispersed in deionized water and hydrated overnight under stirring. β‐Glucan was prepared at the same concentration in deionized water by continuous stirring at 80°C overnight until completely dissolved. Both pectin and β‐glucan solutions were centrifuged at 8000 × g for 120 min and filtered through a 0.45 µm syringe filter to remove undissolved fibers. For NG fabrication, ZnCl_2_ was added dropwise to pectin solution and stirred for 2 h. Astatine, dissolved in PEG solution (50 w/v%), was mixed with PFOA and then added dropwise to the pectin ZnCl_2_ mixture, followed by stirring at RT for 2 h. The resulting suspension was subsequently added dropwise to the β‐glucan solution and stirred at RT overnight. The component ratios of ZnCl_2_, pectin, PFOA, and β‐glucan were listed in Table S1. The final NGs were dialyzed against PBS (pH 7.4, MWCO  =  100  kDa) for 24 h to remove residual monomers and unencapsulated components.

### 4.11 Characterization of NGs

Hydrodynamic size and zeta potential of the obtained NGs were determined using a Malvern Zetasizer, and morphology was examined by transmission electron microscopy (TEM). Encapsulation efficiency (EE) of Ast in NGs was determined by ultrafiltration followed by HPLC analysis of unencapsulated Ast. Ast was quantified using a Shimadzu LC‐30A HPLC system (Japan) fitted with a C18 column (5 µm, 4.6 mm × 25 cm). Chromatographic separation was carried out with methanol/water (30:70, v/v) as the mobile phase at a flow rate of 1 mL min^−1^. EE (%) was calculated using the equation: EE (%) = (total weight of Ast in feed‐weight of unencapsulated Ast) × 100% / total weight of Ast in feed.

#### 4.11.1 Stability of NGs Under pH Conditions and Bile Salts Environment Mimicking the GI Tract

NG suspensions (0.5 mL) were placed in a dialysis bag (MWCO = 100 kDa) and initially immersed in 10 mL pH 7.4 buffer at 37°C for 1 h under 100 rpm shaking to simulate storage conditions. The pH was then adjusted to 1.2 using 1 M HCl to mimic gastric fluid, followed by incubation at 37°C for 1 h under 100 rpm shaking. Subsequently, the pH was raised to 6.8 using 1 M NaOH to simulate intestinal fluid and further incubated at 37°C for 12 h under 100 rpm shaking. Particle size and released Ast were determined at each stage. Similarly, NG suspensions in a dialysis bag were immersed in 4% bile salts for detection.

#### 4.11.2 Stability of NGs in Mouse GI Fluids

Young C57BL/6 mice were fasted for 12 h and euthanized by CO_2_ asphyxiation. The GI tract was dissected into the stomach, small intestine (duodenum, jejunum, and ileum), and large intestine (cecum and colon). Each segment was placed in a Petri dish on ice, opened longitudinally, and the luminal contents were gently collected by scraping mechanically with a sterile spatula into microcentrifuge tubes. Samples were cleared of large debris by centrifugation (4°C, 1200 × g, 10 min) and diluted 10 fold in PBS for subsequent experiments. Various NGs were placed in dialysis bags as mentioned above and immersed in stomach fluid at 37°C for 1 h with shaking at 100 rpm, followed by incubation in small intestinal fluid for 2 h and in large intestinal fluid for 12 h under the same conditions. Particle size and Ast release were measured at each stage.

#### 4.11.3 Stability of NGs in β‑Glucanase

Prior to evaluating the release of Ast from NGs in β‑glucanase, the enzymatic activity of β‑glucanase in intestinal fluid was measured. Intestinal fluid from duodenum, jejunum, ileum, cecum and colon was collected individually as described above. Each fluid was diluted 10 fold in PBS and analyzed using a β‑glucanase activity assay kit following the manufacturer's instructions. Subsequently, different NGs were placed in dialysis bags and immersed in a pH 6.8 solution containing β‑glucanase at a level of 10 U mL^−1^. After 6 h incubation at 37°C with shaking at 100 rpm, the outer solution was analyzed by HPLC to quantify the amount of released Ast.

#### 4.11.4 β‑Glucanase Responsive Ast Release

A dialysis assay was employed to characterize the in vitro release profile of Ast. Briefly, 0.5 mL of Ast@NGs or F_2_‐Ast@NGs was transferred into a dialysis bag and incubated against 10 mL of PBS (pH 7.4) supplemented with β‐glucanase (10 U mL^−1^). At predetermined time intervals, an aliquot of the released 0.2 mL solution was taken out and replenished with an equal volume of fresh β‑glucanase solution. The Ast concentration in each aliquot was determined by HPLC.

#### 4.11.5 Mucin Affinity Assay

NGs suspensions (200 µL) were mixed with 1 mL 0.5% (w/v) porcine mucin solution, and then vortexed and incubated at 37°C for 0.5 h with shaking at 100 rpm. After centrifugation (4°C, 15000 × g, 10 min), the precipitates were washed twice with cold PBS and treated with methanol for 10 min to disrupt NGs. AST content was measured by HPLC. Furthermore, NGs were mixed with 0.001% (w/v) porcine mucin solution and incubated at RT for 10 min, and the hydrodynamic diameter was determined by DLS.

#### 4.11.6 Multiple Particle Tracking (MPT) in Mucus

The Brownian motion of NGs in the 50% intestinal mucus (v/v, diluted with PBS) was investigated by the MPT method. Cy5‐labeled NGs (20 µL) were mixed with 200 µL of mucus and transferred to microwells (Ф = 20 mm). After 0.5 h of equilibrium at 37°C, particle motion was recorded at 5 frames s^−1^ by confocal laser scanning microscopy (CLSM). Tracks were analyzed with Imaris software to acquire the Brownian movement, and the mean squared displacement (MSD) was calculated at different lag times (τ).

#### 4.11.7 Mucus Penetration of NGs

Mouse colons were collected from young C57BL/6 mice and opened longitudinally. The colon was then cut into small pieces around 4 × 4 mm and put into a Petri dish on ice. Cy5‐labeled NGs (20 µL) were dropped into each colon piece and incubated at 37°C for 2 h. Subsequently, colon pieces were washed three times with PBS gently and imaged using an In Vivo Imaging System (IVIS) (Ex = 620 nm, Em = 670 nm) to detect the residual NGs on the colon.

A transwell system was further employed to assess the penetration capacity of NGs. Briefly, HT29 cells were seeded in the basal chamber, while purified intestinal mucus was evenly coated onto the polycarbonate membrane of the apical chamber. FITC‐labeled NGs were then added to the apical compartment and incubated at 37°C with gentle agitation at 50 rpm. After 6 h, fluorescence images of HT29 cells were captured under a fluorescence microscope, and fluorescence intensity (FI) of the medium in the basal chamber was quantified to evaluate NG transport across the mucus barrier.

### 4.12 NGs Distribution in Mouse Intestine

Young colitis mice were orally gavaged with Cy5‐labeled NGs at a dose equivalent to 20 mg Ast kg^−1^. At 3, 6, 12, 24, and 48 h post‐administration, the entire intestine was harvested and visualized using an IVIS system (Ex = 620 nm, Em = 670 nm). The intestine was divided into five sections (duodenum, jejunum, ileum, cecum, and colon). The jejunum was defined as the proximal 40% of the small intestine (beginning at the ligament of Treitz), and the ileum as the distal 60% (ending at the ileocecal valve). FI in each intestinal section was quantified using Living Imaging software. For histological analysis, colon sections were stained with DAPI for 10 min and imaged using CLSM. In addition, young colitis mice were orally administered Cy5‐labeled NGs, followed by rectal instillation of 100 µL FITC‐labeled wheat germ agglutinin (1 µg mL^−1^). Whole‐mount colon tissues were excised, mounted directly on glass slides, and imaged using CLSM. Three‐dimensional tomographic imaging of the colon was performed at 300 µm optical section intervals.

### 4.13 Therapeutic Efficacy of NGs Against Colitis Mouse Model

Young and aged C57BL/6 mice were randomly divided into 10 groups (*n* = 6): Young healthy, Young colitis, Young Ast, Young Ast@NG, Young F_2_‐Ast@NG, Aged healthy, Aged colitis, Aged Ast, Aged Ast@NGs and Aged F_2_‐Ast@NGs. All mice were orally gavaged with different formulations at a dose equivalent to 20 mg Ast kg^−1^ every 2 days from day 2 to day 10 for a total of 5 doses. On day 11, mice were euthanized, and the spleen was weighed while colon tissues were harvested for H&E staining. Additional colon sections were stained with DAPI and anti‐Claudin‐1, and FI of Claudin‐1 in immunofluorescent sections was quantified using ImageJ software. Cytokine levels of IL‐6, IL‐1β, TNF‐α, IL‐17A, IL‐10, and TGF‐β in the colon were quantified using ELISA kits. In parallel, the mRNA levels of IL‐6, IL‐1β, TNF‐α, IL‐17A, IL‐10 and TGF‐β were evaluated with the qRT‐PCR StepOnePlus Real‐Time PCR system. The primers used for mRNA level analysis were listed in Table S2. Moreover, colonic lamina propria cells were stained with Live/Dead solution, anti‐mouse CD45, anti‐mouse CD4, anti‐mouse PD‐1, SA‐β‐gal, anti‐mouse P21, anti‐mouse CD25, anti‐mouse Foxp3, anti‐mouse IL‐17A, anti‐mouse F4/80, anti‐mouse TNF‐α and anti‐mouse CD11c for FC analysis.

### 4.14 Quantification of Short‐Chain Fatty Acids (SCFAs) in Feces

Cecal fecal samples were first homogenized and acidified with hydrochloric acid, followed by extraction with ethyl acetate. After thorough mixing, samples were centrifuged at 15,000 × g for 10 min at 4°C, and the resulting organic phase was dried over anhydrous sodium sulfate. Standard curves were generated using acetate, propionate, butyrate, and valerate. SCFA concentrations were determined on an Agilent 7820A gas chromatograph (USA) equipped with a flame ionization detector and a PEG‐20M capillary column (0.32 mm × 30 m, 0.5 µm). The injector and detector were both maintained at 250°C. Nitrogen was used as the carrier gas at 1.2 mL min^−1^, with an injection volume of 1 µL. The oven was initially held at 100°C for 3.5 min, then ramped to 250°C at 8°C min^−1^ and maintained at the final temperature for 1 min.

### 4.15 16S rDNA Sequencing of Intestinal Microbiota

Microbial DNA was isolated from fecal samples using a Fecal Genome DNA Extraction Kit, and DNA concentration was measured with a Qubit fluorometer (Invitrogen, USA). The V3‐V4 region of bacterial 16S rDNA was amplified using the 341F and 805R primer pair, and PCR products were purified with AMPure XT beads (Beckman Coulter, USA). Purified amplicons were normalized, pooled at equal molar ratios, and subjected to paired‐end sequencing on an Illumina NovaSeq 6000 platform (PE250; LC‐Bio, China). Sequence reads were processed sequentially by adapter and primer removal using cutadapt v1.9, read merging with FLASH v1.2.8, quality trimming with fqtrim v0.94, and chimera filtering using Vsearch v2.3.4. Amplicon sequence variants were subsequently inferred through DADA2, and taxonomic annotation was performed using the QIIME2 feature‐classifier against the SILVA and NT‐16S reference databases. Alpha‐ and beta‐diversity analyses were conducted in QIIME2. Differences in taxonomic abundance were evaluated using the Wilcoxon rank‐sum test and LEfSe analysis, with an LDA score ≥ 3.0 and *P* < 0.05 considered significant.

### 4.16 Therapeutic Efficacy of Empty NGs and Comparison with a Clinically Approved IBD Drug

Aged C57BL/6 mice were randomly divided into 6 groups (*n* = 6): Healthy, Colitis, Ast, NGs, 5‐ASA + F_2_‐Ast@NGs. Ast and F_2_‐Ast@NGs mice were orally gavaged at an equivalent Ast dose of 20 mg kg^−1^ every 2 days from day 2 to day 10 for a total of 5 doses. 5‐ASA group received oral gavage of 5‐ASA at 100 mg kg^−1^ once daily from day 2 to day 10 for a total of 9 doses. On day 11, mice were euthanized, and colon tissues were harvested. Colonic lamina propria cells were stained with Live/Dead solution, anti‐mouse CD45, anti‐mouse CD4, anti‐mouse PD‐1, SA‐β‐gal, anti‐mouse CD28, and anti‐mouse CD27 for FC analysis.

### 4.17 Ex Vivo Characterization of Senescence‐Associated and Non‐Senescence‐Associated CD4^+^ T Cells

Splenocytes were isolated from aged colitis mice as described above. CD4^+^ T cells were purified using a CD4^+^ T Cell Isolation Kit and stained with anti‐mouse CD27 and anti‐mouse CD28 antibodies. CD4^+^ CD27^−^ CD28^−^ T cells were sorted by FC and defined as senescence‐associated CD4^+^ T cells, whereas the remaining CD4^+^ T cells were collected as non‐senescence‐associated CD4^+^ T cells. Sorted cells were stimulated with plate‐bound anti‐CD3 and anti‐CD28 antibodies in the medium supplemented with 100 IU mL^−1^ IL‐2 and cultured in the presence of Ast (500 ng mL^−1^) for 2 days. Subsequently, cells were harvested and stained with Live/Dead dye, anti‐mouse PD‐1, anti‐mouse CD4, anti‐mouse CD25, and anti‐mouse Foxp3 for FC analysis. Cell viability was assessed using the Alamar Blue assay kit according to the manufacturer's instructions.

### 4.18 In Vivo and Ex Vivo Evaluation of PD‐1 Blockade or Activation in Senescence‐Associated CD4^+^ T Cells

Aged C57BL/6 mice were randomly assigned to 5 groups (*n* = 6): Healthy, Colitis, blocking aPD‐1, F_2_‐Ast@NGs and blocking aPD‐1 + F_2_‐Ast@NGs. Mice in the aPD‐1 and combination groups received blocking aPD‐1 antibody via intravenous injection at 200 µg per mouse every other day from day 2 to day 10, for a total of five administrations. On day 11, mice were euthanized, and colons were collected. Colonic lamina propria cells were stained with Live/Dead solution, anti‐mouse CD45, anti‐mouse CD4, anti‐mouse CD27, and anti‐mouse CD28 for FC analysis.

CD4^+^ T cells were isolated from the spleens of aged mice and stimulated on plates pre‐coated with anti‐CD3 and anti‐CD28 antibodies in complete medium supplemented with IL‐2 (100 IU mL^−1^). Cells were cultured in the presence of Ast (500 ng mL^−1^) or plate‐bound agonistic aPD‐1 for 2 days. Subsequently, cells were harvested and stained with Live/Dead dye, anti‐mouse CD27 and anti‐mouse CD28 antibodies for FC analysis.

### 4.19 Therapeutic Efficacy of NGs Against Foxp3^+^ T_regs_‐Depleted Colitis Mouse Model

To investigate the functional role of Foxp3^+^ T_regs_, Foxp3‐IRES‐DTR aged mice were used, enabling in vivo complete depletion of Foxp3^+^ T_regs_ by daily intravenous administration of diphtheria toxin (2 µg/kg) until the study endpoint [59]. Aged Foxp3‐IRES‐DTR mice were randomly divided into 2 groups (*n* = 6): Colitis and F_2_‐Ast@NGs. For comparison, aged C57BL/6 mice were randomly divided into 3 groups (*n* = 6): Healthy, Colitis, and F_2_‐Ast@NGs. All mice were orally gavaged with different formulations at a dose equivalent to 20 mg Ast kg^−1^ every 2 days from day 2 to 10, for a total of 5 times. On day 11, mice were euthanized, and colon tissues were harvested for H&E sections.

### 4.20 Adoptive Transfer of Regulatory Cells

Aged C57BL/6 donor mice were orally gavaged with Ast@NGs or F_2_‐Ast@NGs every two days from day ‐9 to day 1. On day 2, CD4^+^ CD25^+^ T cells were isolated from colonic tissues using the EasySep Mouse CD4^+^ CD25^+^ T Cell Isolation Kit according to the manufacturer's protocol. The resulting cells were intravenously transferred into aged colitis recipient mice at 1 × 10^6^ cells per mouse. DAI was monitored, and colon length was recorded at the endpoint. Colonic sections were subjected to immunofluorescence staining for DAPI, CD4, and Foxp3. In parallel, levels of IL‐6, IL‐1β, TNF‐α, IFN‐γ, IL‐10, and TGF‐β in colon tissues were quantified.

To verify the contribution of Foxp3^+^ T_regs_ to the observed protection, Foxp3‐IRES‐DTR aged mice were used as donors. These mice received the same Ast@NGs or F_2_‐Ast@NGs pretreatment regimen, followed by depletion of Foxp3^+^ cells via daily intravenous diphtheria toxin administration (2 µg/kg). CD4^+^ CD25^+^ T cells were then isolated and transferred into aged UC recipients as described above, after which DAI and colon length were assessed.

### 4.21 Hemolysis Assay

Ast@NGs and F_2_‐Ast@NGs were dispersed in PBS at concentrations ranging from 10 to 200 µg mL^−1^. Whole blood (0.4 mL) was collected from C57BL/6 mice and mixed with 100 µL of 1% sodium heparin for anticoagulation. The sample was diluted with 4 mL PBS and centrifuged at 4°C, 10 000 g for 5 min. After five PBS washes, the obtained red blood cells (RBCs) were resuspended in 4 mL PBS. For hemolysis assessment, 200 µL of RBC suspension was combined with 800 µL of each NG formulation in 1.5 mL microcentrifuge tubes. PBS and deionized water were included as the negative and positive controls, respectively. After incubation at room temperature for 4 h, samples were centrifuged at 10 000 × g for 3 min at 4°C. The absorbance of the resulting supernatants was recorded at 577 nm using 655 nm as the reference wavelength. Hemolysis was then determined using the following equation: Hemolysis (%) = (Experiment − Negative) × 100% / (Positive − Negative) [60].

### 4.22 In Vivo Safety Evaluation of NGs

Aged C57BL/6 mice were randomly divided into 4 groups (*n* = 6): PBS, Ast, Ast@NGs, and F_2_‐Ast@NGs. Ast was suspended in 0.5% CMC‐Na, and NGs were dissolved in PBS. C57BL/6 mice were orally gavaged with different formulations at a dose equivalent to 100 mg Ast kg^−1^ every 2 days from day 2 to 10, with a total of 5 doses. On day 11, blood was collected from mice in each group for liver function (alanine aminotransferase and aspartate aminotransferase), kidney function (uric acid and creatinine), and complete blood count analysis. After euthanizing mice, major organs of the heart, liver, spleen, lung, kidney, duodenum, jejunum, ileum, and colon were harvested, fixed, and examined by H&E staining for any pathological abnormalities.

In addition, a three‐week repeated‐dose safety study was performed to evaluate the safety of prolonged administration. Aged C57BL/6 mice received the indicated formulations by oral gavage every 2 days for a total of 10 doses. Body weight was monitored throughout the study, and serum biochemical parameters, complete blood counts, and histological changes in different intestinal segments were evaluated at the end of the study.

### 4.23 Statistical Analysis

All data are presented as mean ± SD, and statistical analysis was performed using GraphPad Prism software (version 10.1). Comparisons between two groups were made using unpaired two‐tailed t‐tests, while One‐way or Two‐way ANOVA followed by Tukey's post hoc test was used for multiple group comparisons.

## Author Contributions

**Lei Zhao**: conceptualization, methodology, data curation, investigation, formal analysis, funding acquisition, writing – original draft. **Xinyun Zhang**: methodology, data curation, investigation, formal analysis, funding acquisition, writing – original draft. **Qian Ren**: data curation, software, investigation, writing – original draft, formal analysis, validation. **Xue Song**: investigation, validation. **Meifang Zhao**: investigation, data curation. **Rongshen Wang**: methodology. **Haocheng Du**: investigation. **Yan Chen**: methodology, investigation. **Peigen Xu**: investigation. **Jiangyun Liu**: conceptualization. **Jian Zhang**: writing – review and editing, funding acquisition, conceptualization, project administration, resources. **Jingjing Ma**: writing – review and editing, project administration, conceptualization, methodology, resources, funding acquisition, supervision.

## Funding

This work was supported by collaborative project with Nantong Saikon Biotechnology Co., Ltd. (H250954), State Key Laboratory of Advanced Drug Delivery and Release Systems (DSQZ‐QN‐20250102), National Natural Science Foundation of China (82400241), Suzhou Medical College‐QiLu Medical Research Program of Soochow University (24QL200212), Research Pre‐research Fund Project of the Second Affiliated Hospital of Soochow University (SDFEYBS2307), the 2025 Special Research Project, Capacity Building and Continuing Education Center of the National Health Commission of China (GWJJZX20251007061), the Gusu Health Talent Program of Suzhou (GSWS2025062), the open fund of the National Key Laboratory of Advanced Drug Formulations for Overcoming Delivery Barriers, Young Talents Program of China National Nuclear Corporation, Jiangsu Province Engineering Research Center of Molecular Target Therapy and Companion Diagnostics in Oncology, Suzhou Vocational Health College (SGK1202412), The Natural Science Foundation of the Jiangsu Higher Education Institutions of China (24KJB350009), the Opening Foundation of State Key Laboratory of Neurology and Oncology Drug Development(SKLSIM‐20251432) and Suzhou Science and Education for Health Empowerment Project (ZDXM2025010).

## Conflicts of Interest

The authors declare no conflict of interest.

## Supporting information




**Supporting File**: advs78123‐sup‐0001‐SuppMat1.docx.

## Data Availability

The data that support the findings of this study are available from the corresponding author upon reasonable request.

## References

[1] S. Vieujean , V. Jairath , L. Peyrin‐Biroulet , et al., “Understanding the Therapeutic Toolkit for Inflammatory Bowel Disease,” Nature Reviews Gastroenterology & Hepatology 22 (2025): 371–394, 10.1038/s41575-024-01035-7. 39891014

[2] L. Hracs , J. W. Windsor , J. Gorospe , et al., “Global Evolution of Inflammatory Bowel Disease across Epidemiologic Stages,” Nature 642 (2025): 458–466, 10.1038/s41586-025-08940-0. PMC12158780 40307548

[3] V. Solitano , C. N. Bernstein , I. Dotan , et al., “Shaping the Future of Inflammatory Bowel Disease: a Global Research Agenda for Better Management and Public Health Response,” Nature Reviews Gastroenterology & Hepatology 22 (2025): 438–452, 10.1038/s41575-025-01063-x. 40263433

[4] S. C. Ng , H. Y. Shi , N. Hamidi , et al., “Worldwide Incidence and Prevalence of Inflammatory Bowel Disease in the 21st Century: a Systematic Review of Population‐based Studies,” Lancet 390 (2017): 2769–2778, 10.1016/S0140-6736(17)32448-0. 29050646

[5] S. Singh , G. J. Poulsen , T. H. Bisgaard , L. Bonfils , and T. Jess , “Epidemiology of Elderly Onset IBD: a Nationwide Population‐Based Cohort Study,” Clinical Gastroenterology and Hepatology 23 (2025): 1204–1215, 10.1016/j.cgh.2024.08.011. PMC12186701 39209204

[6] S. Coward , F. Clement , E. I. Benchimol , et al., “Past and Future Burden of Inflammatory Bowel Diseases Based on Modeling of Population‐Based Data,” Gastroenterology 156 (2019): 1345–1353, 10.1053/j.gastro.2019.01.002. 30639677

[7] T. S. Ghosh , F. Shanahan , and P. W. O'Toole , “The Gut Microbiome as a Modulator of Healthy Ageing,” Nature Reviews Gastroenterology & Hepatology 19 (2022): 565–584, 10.1038/s41575-022-00605-x. PMC9035980 35468952

[8] H. Jasper , “Intestinal Stem Cell Aging: Origins and Interventions,” Annual Review of Physiology 82 (2020): 203–226, 10.1146/annurev-physiol-021119-034359. 31610128

[9] S. Singh , B. S. Boland , T. Jess , and A. A. Moore , “Management of Inflammatory Bowel Diseases in Older Adults,” Lancet Gastroenterol Hepatol 8 (2023): 368–382, 10.1016/S2468-1253(22)00358-2. 36669515

[10] Y. Jing , Q. Wang , F. Bai , et al., “Age‐related Alterations in Gut Homeostasis Are Microbiota Dependent,” Npj Biofilms Microbomes 11 (2025): 51, 10.1038/s41522-025-00677-y. PMC11937415 40133348

[11] J. J. V. Branca , M. Gulisano , and C. Nicoletti , “Intestinal Epithelial Barrier Functions in Ageing,” Ageing Research Reviews 54 (2019): 100938, 10.1016/j.arr.2019.100938. 31369869

[12] A. N. Ananthakrishnan , G. C. Nguyen , and C. N. Bernstein , “AGA Clinical Practice Update on Management of Inflammatory Bowel Disease in Elderly Patients: Expert Review,” Gastroenterology 160 (2021): 445–451, 10.1053/j.gastro.2020.08.060. 33011177

[13] V. Solitano , A. Facciorusso , T. Jess , et al., “Comparative Risk of Serious Infections with Biologic Agents and Oral Small Molecules in Inflammatory Bowel Diseases: a Systematic Review and Meta‐Analysis,” Clinical Gastroenterology and Hepatology 21 (2023): 907–921, 10.1016/j.cgh.2022.07.032. 35944832

[14] J. J. Goronzy and C. M. Weyand , “Mechanisms Underlying T Cell Ageing,” Nature Reviews Immunology 19 (2019): 573–583, 10.1038/s41577-019-0180-1. PMC7584388 31186548

[15] G. L. H. MM , E. Carrasco , and M. Pérez‐Manrique , “CD4 T Cell Therapy Counteracts Inflammaging and Senescence by Preserving Gut Barrier Integrity,” Science Immunology 10 (2025): adv0985, 10.1126/sciimmunol.adv0985. PMC7618201 40749035

[16] Y. Zhang , J. Xu , Y. Zhang , et al., “Colonic CD4+T Cell Senescence Is Implicated in the Progression of Experimental Colitis in Aged Mice,” Journal of Crohn's and Colitis 18 (2024): I228–I229, 10.1093/ecco-jcc/jjad212.0124.

[17] D. Širvinskas , O. Omrani , J. Lu , et al., “Single‐cell Atlas of the Aging Mouse Colon,” iScience 25 (2022): 104202, 10.1016/j.isci.2022.104202. PMC9035718 35479413

[18] Y. Gu , R. Bartolomé‐Casado , C. Xu , et al., “Immune Microniches Shape Intestinal Treg Function,” Nature 628 (2024): 854–862, 10.1038/s41586-024-07251-0. PMC11041794 38570678

[19] D. Zheng , T. Liwinski , and E. Elinav , “Interaction between Microbiota and Immunity in Health and Disease,” Cellular Respiration 30 (2020): 492–506, 10.1038/s41422-020-0332-7. PMC7264227 32433595

[20] K. Shimatani , Y. Nakashima , M. Hattori , Y. Hamazaki , and N. Minato , “PD‐1+ Memory Phenotype CD4+ T Cells Expressing C/EBPalpha Underlie T Cell Immunodepression in Senescence and Leukemia,” Proceedings of the National Academy of Sciences of the USA 106 (2009): 15807–15812, 10.1073/pnas.0908805106. PMC2739871 19805226

[21] D. A. Mogilenko , O. Shpynov , P. S. Andhey , et al., “Comprehensive Profiling of an Aging Immune System Reveals Clonal GZMK+ CD8+ T Cells as Conserved Hallmark of Inflammaging,” Immunity 54 (2021): 99, 10.1016/j.immuni.2020.11.005. 33271118

[22] Q. Yang , W.‐H. He , L. Xie , et al., “Oral Administration of Astilbin Mitigates Acetaminophen‐induced Acute Liver Injury in Mice by Modulating the Gut Microbiota,” Acta Pharmacologica Sinica 46 (2025): 416–429, 10.1038/s41401-024-01383-9. PMC11747501 39313515

[23] G. Shi , T. Tai , Y. Miao , et al., “The Antagonism Mechanism of Astilbin against Cadmium‐induced Injury in Chicken Lungs via Treg/Th1 Balance Signaling Pathway,” Ecotoxicology and Environmental Safety 277 (2024): 116364, 10.1016/j.ecoenv.2024.116364. 38657461

[24] L. Zhao , Z. Qi , L. Yi , J. Li , and Y. Cui , “The Interaction between Gut Microbiota and Flavonoid Extract From Smilax glabra Roxb. And Its Potent Alleviation of Fatty Liver,” Functional Foods 12 (2021): 7836–7850, 10.1039/d1fo00727k. 34235516

[25] D. Yang and Q.‐F. Zhang , “The Natural Source, Physicochemical Properties, Biological Activities and Metabolism of Astilbin,” Critical Reviews in Food Science and Nutrition 63 (2023): 9506–9518, 10.1080/10408398.2022.2065661. 35442837

[26] Y. Xu , K. Wu , S. Han , et al., “Astilbin Combined with Lipopolysaccharide Induces IL‐10‐producing Regulatory B Cells via the STAT3 Signalling Pathway,” Biomedicine & Pharmacotherapy 129 (2020): 110450, 10.1016/j.biopha.2020.110450. 32768945

[27] Y. Zhang , C. Ding , Y. Cai , et al., “Astilbin Ameliorates Oxidative Stress and Apoptosis in D‐galactose‐induced Senescence by Regulating the PI3K/Akt/M‐TOR Signaling Pathway in the Brains of Mice,” International Immunopharmacology 99 (2021): 108035, 10.1016/j.intimp.2021.108035. 34435579

[28] T.‐T. Di , Z.‐T. Ruan , J.‐X. Zhao , et al., “Astilbin Inhibits Th17 Cell Differentiation and Ameliorates Imiquimod‐induced Psoriasis‐like Skin Lesions in BALB/c Mice via Jak3/Stat3 Signaling Pathway,” International Immunopharmacology 32 (2016): 32–38, 10.1016/j.intimp.2015.12.035. 26784569

[29] Q.‐F. Meng , Z. Zhang , and Y.‐J. Wang , “Astilbin Ameliorates Experimental Autoimmune Myasthenia Gravis by Decreased Th17 Cytokines and Up‐regulated T Regulatory Cells,” Journal of Neuroimmunology 298 (2016): 138–145, 10.1016/j.jneuroim.2016.07.016. 27609287

[30] S. Ding , G. Lu , B. Wang , et al., “Astilbin Activates the Reactive Oxidative Species/PPARγ Pathway to Suppress Effector CD4+ T Cell Activities via Direct Binding with Cytochrome P450 1B1,” Frontiers in Pharmacology 13 (2022): 848957, 10.3389/fphar.2022.848957. PMC9150850 35652039

[31] X.‐H. Sun , H. Zhang , X.‐P. Fan , and Z.‐H. Wang , “Astilbin Protects against Carbon Tetrachloride‐Induced Liver Fibrosis in Rats,” Pharmacology 106 (2021): 323–331, 10.1159/000514594. 33780953

[32] B. Golisch , Z. Lei , K. Tamura , and H. Brumer , “Configured for the Human Gut Microbiota: Molecular Mechanisms of Dietary β‐Glucan Utilization,” ACS Chemical Biology 16 (2021): 2087–2102, 10.1021/acschembio.1c00563. 34709792

[33] S. R. Beeren , C. E. Christensen , H. Tanaka , M. G. Jensen , I. Donaldson , and O. Hindsgaul , “Direct Study of Fluorescently‐labelled Barley β‐glucan Fate in an in Vitro human Colon Digestion Model,” Carbohydrate Polymers 115 (2015): 88–92, 10.1016/j.carbpol.2014.08.056. 25439872

[34] S. A. Hughes , P. R. Shewry , G. R. Gibson , B. V. McCleary , and R. A. Rastall , “In Vitro Fermentation of Oat and Barley Derived Beta‐glucans by human Faecal Microbiota,” FEMS Microbiology Ecology 64 (2008): 482–493, 10.1111/j.1574-6941.2008.00478.x. 18430007

[35] M. Khotimchenko , “Pectin Polymers for Colon‐targeted Antitumor Drug Delivery,” International Journal of Biological Macromolecules 158 (2020): 1110–1124, 10.1016/j.ijbiomac.2020.05.002. 32387365

[36] H.‐Y. Tang , Z. Fang , and K. Ng , “Dietary fiber‐based Colon‐targeted Delivery Systems for Polyphenols,” Trends in Food Science & Technology 100 (2020): 333–348, 10.1016/j.tifs.2020.04.028.

[37] Y. Fukushima , R. Ueno , N. Minato , and M. Hattori , “Senescence‐associated T Cells in Immunosenescence and Diseases,” International Immunology 37 (2025): 143–152, 10.1093/intimm/dxae056. 39320393

[38] A. H. Sharpe and K. E. Pauken , “The Diverse Functions of the PD1 Inhibitory Pathway,” Nature Reviews Immunology 18 (2018): 153–167, 10.1038/nri.2017.108. 28990585

[39] J. Kohli , B. Wang , S. M. Brandenburg , et al., “Algorithmic Assessment of Cellular Senescence in Experimental and Clinical Specimens,” Nature Protocols 16 (2021): 2471–2498, 10.1038/s41596-021-00505-5. PMC8710232 33911261

[40] H. Liang , J. Jiang , J. Miao , et al., “A Biomimetic Sweeping Microrobot for Active Therapy of Ulcerative Colitis,” Advanced Materials 37 (2025): 2402579, 10.1002/adma.202402579. 40072036

[41] T. Kobayashi , B. Siegmund , and C. Le Berre , “Ulcerative Colitis,” Nature Reviews Disease Primers 6 (2020): 74, 10.1038/s41572-020-0205-x. 32913180

[42] C.‐P. J. Wang , M. J. Byun , and S.‐N. Kim , “Biomaterials as Therapeutic Drug Carriers for Inflammatory Bowel Disease Treatment,” Journal of Controlled Release 345 (2022): 1–19, 10.1016/j.jconrel.2022.02.028. 35227764

[43] D. Ramanan , A. Pratama , Y. Zhu , et al., “Regulatory T Cells in the Face of the Intestinal Microbiota,” Nature Reviews Immunology 23 (2023): 749–762, 10.1038/s41577-023-00890-w. 37316560

[44] H. Tsukamoto , K. Clise‐Dwyer , and G. E. Huston , “Age‐associated Increase in Lifespan of Naive CD4 T Cells Contributes to T‐cell Homeostasis but Facilitates Development of Functional Defects,” Proceedings of the National Academy of Sciences of the USA 106 (2009): 18333–18338, 10.1073/pnas.0910139106. PMC2759371 19815516

[45] L. Haynes , S. M. Eaton , E. M. Burns , T. D. Randall , and S. L. Swain , “CD4 T Cell Memory Derived From Young Naive Cells Functions Well into Old Age, but Memory Generated From Aged Naive Cells Functions Poorly,” Proceedings of the National Academy of Sciences of the USA 100 (2003): 15053–15058, 10.1073/pnas.2433717100. PMC299903 14657384

[46] T. Korn , E. Bettelli , M. Oukka , and V. K. Kuchroo , “IL‐17 and Th17 Cells,” Annual Review of Immunology 27 (2009): 485–517, 10.1146/annurev.immunol.021908.132710. 19132915

[47] E. Bettelli , Y. J. Carrier , W. D. Gao , et al., “Reciprocal Developmental Pathways for the Generation of Pathogenic Effector TH17 and Regulatory T Cells,” Nature 441 (2006): 235–238, 10.1038/nature04753. 16648838

[48] E. R. Mann , Y. K. Lam , and H. H. Uhlig , “Short‐chain Fatty Acids: Linking Diet, the Microbiome and Immunity,” Nature Reviews Immunology 24 (2024): 577–595, 10.1038/s41577-024-01014-8. 38565643

[49] H. Ju , Y. Zhou , W. Wei , et al., “Ageing‐associated Gut Dysbiosis Deteriorates Mouse Cognition,” Acta Biochimica et Biophysica Sinica 57 (2025): 1234–1243, 10.3724/abbs.2024217. PMC12368526 39943805

[50] X. You , U. C. Dadwal , M. E. Lenburg , M. A. Kacena , and J. F. Charles , “Murine Gut Microbiome Meta‐analysis Reveals Alterations in Carbohydrate Metabolism in Response to Aging,” mSystems 7 (2022): 0124821, 10.1128/msystems.01248-21. PMC9040766 35400171

[51] K. Cai , X.‐Y. Cao , F. Chen , et al., “Xianlian Jiedu Decoction Alleviates Colorectal Cancer by Regulating Metabolic Profiles, Intestinal Microbiota and Metabolites,” Phytomedicine 128 (2024): 155385, 10.1016/j.phymed.2024.155385. 38569292

[52] J. Hu , Y. Mei , H. Zhang , et al., “Ameliorative Effect of an Acidic Polysaccharide From Phellinus linteus on Ulcerative Colitis in a DSS‐induced Mouse Model,” International Journal of Biological Macromolecules 265 (2024): 130959, 10.1016/j.ijbiomac.2024.130959. 38499127

[53] P.‐A. Jansson , D. Curiac , and I. Lazou Ahrén , “Probiotic Treatment Using a Mix of Three Lactobacillus Strains for Lumbar Spine Bone Loss in Postmenopausal Women: a Randomised, Double‐blind, Placebo‐controlled, Multicentre Trial,” Lancet Rheumatol 1 (2019): e154–e162, 10.1016/S2665-9913(19)30068-2. 38229392

[54] M. T. Sorbara , E. R. Littmann , E. Fontana , et al., “Functional and Genomic Variation between Human‐Derived Isolates of Lachnospiraceae Reveals Inter‐ and Intra‐Species Diversity,” Cell Host Microbe 28 (2020): 134–146, 10.1016/j.chom.2020.05.005. PMC7351604 32492369

[55] Q. Hu , J. Li , T. Wang , X. Xu , Y. Duan , and Y. Jin , “Polyphenolic Nanoparticle‐Modified Probiotics for Microenvironment Remodeling and Targeted Therapy of Inflammatory Bowel Disease,” ACS Nano 18 (2024): 12917–12932, 10.1021/acsnano.4c00830. 38720520

[56] J. Li , J. Xu , X. Guo , et al., “Odoribacter Splanchnicus‐A Next‐Generation Probiotic Candidate,” Microorganisms 13 (2025): 815, 10.3390/microorganisms13040815. PMC12029356 40284651

[57] Y. Chen , Y.‐C. Su , and S. R. Roffler , “Polyethylene Glycol Immunogenicity in Nanomedicine,” Nature Reviews Bioengineering 3 (2025): 742–760, 10.1038/s44222-025-00321-6.

[58] Y. Ju , J. M. Carreño , V. Simon , K. Dawson , F. Krammer , and S. J. Kent , “Impact of Anti‐PEG Antibodies Induced by SARS‐CoV‐2 mRNA Vaccines,” Nature Reviews Immunology 23 (2023): 135–136, 10.1038/s41577-022-00825-x. PMC9764299 36539526

[59] L. Zhao , Z. Gao , Z. Yuan , et al., “DC‐CD4 Bispecific Tolerogenic Nanovesicles Induce Antigen‐specific Regulatory T Cells and Ameliorate Collagen‐induced Arthritis in Mice,” Nature Communication 17 (2026): 4170, 10.1038/s41467-026-70898-y. PMC13153181 41851116

[60] W. Deng , J. Chen , X. Wang , et al., “Paravertebrally‐Injected Multifunctional Hydrogel for Sustained Anti‐Inflammation and Pain Relief in Lumbar Disc Herniation,” Advanced Healthcare Materials 13 (2024): 2401227, 10.1002/adhm.202401227. 38979866

