# Supplementary material for: Oral Astilbin Nanogels Modulate Senescence‐Associated CD4+ T Cells and Promote Treg Induction in Aged Mice with Colitis
Source: Adv Sci (Weinh). 2026 Oct 18:e78123. Online ahead of print. doi: 10.1002/advs.78123 (PMC13676816; doi:10.1002/advs.78123)
Supplement: Supplementary file 1 [file ADVS-9999-e78123-s001.docx]

Supporting Information

**Oral Astilbin Nanogels Modulate Senescence-Associated Features of Colonic CD4⁺ T Cells and Promote T_reg_ Induction in Aged Mice with Colitis**

Lei Zhao^†,^* Xinyun Zhang^†^, Qian Ren^†^, Xue Song, Meifang Zhao, Rongshen Wang, Haocheng Du, Yan Chen, Peigen Xu, Jiangyun Liu, Jian Zhang*, Jingjing Ma*

This file includes:

Figures S1 to S28

Table S1 to S2

**Fig. S1. Comparison between young and aged colitis mice.** (A) Representative fecal samples following occult blood testing. (B) Curve chart variations of murine body weight across time, normalized to the day 0 body weight percentage (*n* = 6). (C) Concentration of IL-17A in colon tissues (*n* = 6). (D) Representative H&E stained colon sections and quantification of histological scores (*n* = 6) (Scale bar = 100 μm). (E) Representative histogram and quantitative analysis of P21^+^ in CD4^+^ T cells in colonic lamina propria cells (*n* = 6). (F) Representative FC plots and quantitative analysis of CD4^+^ CD25^+^ Foxp3^+^ T cells in colonic lamina propria cells (*n* = 6). Data are presented as mean ± SD, and analyzed by Two-way ANOVA with Tukey’s post-test (B) or One-way ANOVA with Tukey’s post-test (C-F). ns represents not significant, **P* < 0.05, ***P* < 0.01, ****P* < 0.001 and *****P* < 0.0001.

**Fig. S2.** Role of colonic CD4^+^ T cells in the pathogenesis of colitis mice. (A) Experimental procedure of the adoptive transfer of colonic CD4^+^ T cells into young colitis mice. (B) Representative fecal samples following occult blood testing. (C) Curve chart variations of murine body weight across time, normalized to the day 0 body weight percentage (*n* = 6). (D) Curve chart analysis of DAI (*n* = 6). (E) Representative images of colons and corresponding quantification of colon length (*n* = 6). (F) Concentration of IL-17A in colonic tissues (*n* = 6). Data are presented as mean ± SD, and analyzed by Two-way ANOVA with Tukey’s post-test (C, D) or One-way ANOVA with Tukey’s post-test (E, F). ns represents not significant, **P* < 0.05 and ***P* < 0.01.

**Fig. S3.** *In* *vitro* effects of Ast on PD-1 expression in colonic CD4^+^ T cells isolated from young and aged mice. Data are presented as mean ± SD (*n* = 6), and analyzed by two-tailed unpaired Student’s t test. ns represents not significant, ***P* < 0.01 and *****P* < 0.0001.

**Fig. S4.** *In* *vitro* effects of different concentrations of Ast on PD-1 expression in colonic CD4^+^ T cells isolated from young and aged mice. Data are presented as mean ± SD (*n* = 5), and analyzed by One-way ANOVA with Tukey’s post-test. ns represents not significant, **P* < 0.05.

**Fig. S5.** *In* *vitro* effects of different concentrations of Ast on P21 expression in colonic CD4^+^ T cells isolated from young and aged mice. Data are presented as mean ± SD (*n* = 5), and analyzed by One-way ANOVA with Tukey’s post-test. ns represents not significant, **P* < 0.05.

**Fig. S6.** Representative fecal samples after fecal occult blood testing following free Ast treatment.

**Fig. S7.** Representative histogram and quantitative analysis of P21^+^ in CD4^+^ T cells in colonic lamina propria cells after free Ast treatment. Data are presented as mean ± SD (*n* = 6), and analyzed by One-way ANOVA with Tukey’s post-test. ns represents not significant, **P* < 0.05.

**Fig. S8.** Transcriptomic analysis of free Ast on aged colitis mice. (A) PCA profiles (*n* = 6). (B) Heatmap of differentially expressed anti-inflammatory genes in colon samples (*n* = 6). (C) GO enrichment analysis between colitis and Ast group.

**Fig. S9.** Characterization of NGs by (A) encapsulation efficacy and (B) polydispersity index. Data are presented as mean ± SD (*n* = 3).

**Fig. S10.** Stability of uncoated Ast@NGs and β-glucan-coated NGs under varying pH conditions. (A) Changes in particle size of NGs (*n* = 3). (B) Released content of Ast from NGs (*n* = 3). (C) Cumulative Ast release from NGs (*n* = 3). Data are presented as mean ± SD.

**Fig. S11.** Stability of uncoated Ast@NGs and β-glucan-coated NGs under 4% bile salts. (A) Changes in particle size of NGs (*n* = 3). (B) Released content of Ast from NGs (*n* = 3). Data are presented as mean ± SD.

**Fig. S12.** FI curve of duodenum, jejunum, ileum and cecum at different time points. Data are presented as mean ± SD (*n* = 6).

**Fig. S13.** AUC of colonic FI from 3 to 48 h. Data are presented as mean ± SD (*n* = 6).

**Fig. S14.** Extended results of therapeutic effects of F_2_-Ast@NGs on young and aged colitis mice. (A) Curve chart variations of murine body weight across time (*n* = 6). (B) Ratio of spleen weight to body weight (*n* = 6). (C) Quantitative analysis of claudin-1 from colonic immunoﬂuorescence sections (*n* = 6). Data are presented as mean ± SD, and analyzed by Two-way ANOVA with Tukey’s post-test (A) or One-way ANOVA with Tukey’s post-test (B, C). ns represents not significant, **P* < 0.05, ***P* < 0.01, ****P* < 0.001 and *****P* < 0.0001.

**Fig. S15.** mRNA levels of cytokines including IL-6, IL-1β, TNF-α, IL-17A, IL-10 and TGF-β in colonic tissue from (A) young and (B) aged mice. Data are presented as mean ± SD (*n* = 6), and analyzed by One-way ANOVA with Tukey’s post-test. ns represents not significant, **P* < 0.05, ***P* < 0.01, ****P* < 0.001 and *****P* < 0.0001.

**Fig. S16.** FC analysis of colonic CD4^+^ T cells. Representative FC plots and quantitative analysis of CD4^+^ PD-1^+^ cells in colonic lamina propria cells from (A) young and (B) aged mice (*n* = 6). Representative FC plots of CD25^+^ Foxp3^+^ cells from (C) young and (D) aged mice. Representative FC plots of CD4^+^ IL-17A^+^ cells from (E) young and (F) aged mice. Data are presented as mean ± SD, and analyzed by One-way ANOVA with Tukey’s post-test. ns represents not significant, ***P* < 0.01 and *****P* < 0.0001.

**Fig. S17.** Representative histogram and quantitative analysis of P21^+^ in CD4 T cells in colonic lamina propria cells. Data are presented as mean ± SD (*n* = 6), and analyzed by One-way ANOVA with Tukey’s post-test. ns represents not significant, **P* < 0.05 and ***P* < 0.01.

**Fig. S18.** FC analysis of myeloid cells. Representative FC plots of TNF-α^+^ F4/80^+^ cells in colonic lamina propria cells from (A) young and (B) aged mice. Representative FC plots of TNF-α^+^ CD11c^+^ cells from (C) young and (D) aged mice.

**Fig. S19.** The levels of acetate, propionate, butyrate and valerate in the intestinal from (A) young and (B) aged mice. Data are presented as mean ± SD (*n* = 6), and analyzed by One-way ANOVA with Tukey’s post-test. ns represents not significant, **P* < 0.05, ***P* < 0.01 and *****P* < 0.0001.

**Fig. S20.** Intestinal microorganism diversity of shannon and simpson index (*n* = 3). Data are presented as mean ± SD.

**Fig. S21.** Phylum abundance for (A) *Bacteroidota*, (B) *Firmicutes* and (C) *Proteobacteria.* Data are presented as mean ± SD (*n* = 3), and analyzed by One-way ANOVA with Tukey’s post-test. ns represents not significant, ***P* < 0.01 and ****P* < 0.001.

**Fig. S22.** Therapeutic effects in aged DSS-induced colitis mice. (A) Curve chart variations of murine body weight across time (*n* = 6). (B) Quantitative analysis of CD4^+^ SA-β-gal^+^ T cells in colonic lamina propria cells (*n* = 6). Data are presented as mean ± SD, and analyzed by two-way ANOVA with Tukey’s post-test (A) or one-way ANOVA with Tukey’s post-test (B). ns represents not significant, **P* < 0.05 and ****P* < 0.001.

**Fig. S23.** IL-17A levels in the culture supernatants of CD4⁺ T cells following Ast treatment. Data are presented as mean ± SD (*n* = 3), and analyzed by one-way ANOVA with Tukey’s post-test. ns represents not significant, **P* < 0.05.

**Fig. S24.** Representative images of colons from DSS-induced colitis mice treated with aPD-1 or F_2_-Ast@NGs.

**Fig. S25.** Curve chart variations of murine body weight across time. Data are presented as mean ± SD (*n* = 6), and analyzed by Two-way ANOVA with Tukey’s post-test. ns represents not significant and ***P* < 0.01.

**Fig. S26.** Curve chart analysis of DAI scores of normal donor mice. Data are presented as mean ± SD (*n* = 6), and analyzed by Two-way ANOVA with Tukey’s post-test. ns represents not significant, ***P* < 0.01, ****P* < 0.001 and *****P* < 0.0001.

**Fig. S27.** Serum biochemical and hematological analysis. (A) The levels of alanine aminotransferase (ALT) and aspartate aminotransferase (AST) in serum (*n* = 6). (B) The levels of uric acid (UA) and creatinine (CR) in serum (*n* = 6). (C) The count of white blood cell (WBC), lymphocytes (Lymph), monocytes (Mon), neutrophils (Neut), red blood cells (RBC), platelet (PLT), level of hemoglobin (HGB) and RBC distribution width (*n* = 6). Data are presented as mean ± SD, and analyzed by One-way ANOVA with Tukey’s post-test. ns represents not significant.

**Fig. S28.** Hematological analysis. Data are presented as mean ± SD (*n* = 6), and analyzed by One-way ANOVA with Tukey’s post-test. ns represents not significant.

**Table S1.** Monomer feed ratios used for the preparation of F-Ast@NG.

| Sample | Monomer weight ratio | | | |
| --- | --- | --- | --- | --- |
|  | ZnCl_2_ | Pectin | PFOA | β-glucan |
| Ast@NG | 1 | 10 | 0 | 3 |
| F_1_-Ast@NG | 1 | 10 | 0.14 (1 wt%) | 3 |
| F_2_-Ast@NG | 1 | 10 | 0.29 (2 wt%) | 3 |
| F_3_-Ast@NG | 1 | 10 | 0.43 (3 wt%) | 3 |
| F_4_-Ast@NG | 1 | 10 | 0.58 (4 wt%) | 3 |

**Table S2.** Primer sequences of genes.

| Gene subtype | Oligonucleotide primers (5’-3’) |
| --- | --- |
| IL-6 | F: TAGTCCTTCCTACCCCAATTTCC |
|  | R: TTGGTCCTTAGCCACTCCTTC |
| IL-1β | F: GCAACTGTTCCTGAACTCAACT |
|  | R: ATCTTTTGGGGTCCGTCAACT |
| TNF-α | F: CCCTCACACTCAGATCATCTTCT |
|  | R: GCTACGACGTGGGCTACAG |
| IL-17A | F: CAGACTACCTCAACCGTTCCAC |
|  | R: TCCAGCTTTCCCTCCGCATTGA |
| IL-10 | F: CGGGAAGACAATAACTGCACCC |
|  | R: CGGTTAGCAGTATGTTGTCCAGC |
| TGF-β | R: GGTTCATGTCATGGATGGTGC |
|  | F: TGACGTCACTGGAGTTTGTACGG |
